# Functional limitations in people with multimorbidity and the association with mental health conditions: Baseline data from the Canadian Longitudinal Study on Aging (CLSA)

**DOI:** 10.1371/journal.pone.0255907

**Published:** 2021-08-11

**Authors:** Kathryn Fisher, Lauren E. Griffith, Andrea Gruneir, David Kanters, Maureen Markle-Reid, Jenny Ploeg

**Affiliations:** 1 School of Nursing, McMaster University, Hamilton, Ontario, Canada; 2 Department of Health Research Methods, Evidence, and Impact, McMaster University, Hamilton, Ontario, Canada; 3 Department of Family Medicine, University of Alberta, Edmonton, Alberta, Canada; 4 ICES, Toronto, Ontario, Canada; Universidade Federal de Pelotas, BRAZIL

## Abstract

**Introduction:**

Increasing multimorbidity is often associated with declining physical functioning, with some studies showing a disproportionate impact on functioning when mental health conditions are present. More research is needed because most multimorbidity studies exclude mental health conditions.

**Objectives:**

This study aims to improve our understanding of the association between functional limitation and multimorbidity, including a comparison of those with multimorbidity that includes versus excludes mental health conditions.

**Methods:**

This is a population-based, cross-sectional analysis of data from The Canadian Longitudinal Study on Aging. Functional limitation was defined as the presence of any of 14 activities of daily living (ADLs) or instrumental activities of daily living (IADLs). Multimorbidity, measured by the number of chronic conditions, included mood and anxiety disorders. Logistic regression explored the association between multimorbidity (with and without mental health conditions) and functional limitation. Factor analysis identified common condition clusters to help understand clinical complexity in those with mood/anxiety disorders and the potential influences on functional limitation.

**Results:**

There were 51,338 participants, with a similar proportion of men and women (49% versus 51%) and 42% age 65 years or older. Fifteen percent (15%) had no chronic conditions and 17% had 5+. Ten percent (10%) reported at least one ADL or IADL limitation. Odds ratios (ORs) for functional limitation increased with multimorbidity and were generally higher for those with versus without mental health conditions (e.g., ORs from 1 to 5+ chronic conditions increased 1.9 to 15.8 for those with mood/anxiety disorders versus 1.8 to 10.2 for those without). Factor analysis showed that mood/anxiety conditions clustered with somatic conditions (e.g., migraines, bowel/gastrointestinal disorders).

**Conclusion:**

This study found higher odds of functional limitation for those with multimorbidity that included versus excluded mental health conditions, at all levels of multimorbidity. It highlights the need for concurrent management of mental and physical comorbidities to prevent functional limitations and future decline. This approach is aligned with the NICE clinical assessment and management guidelines for people with multimorbidity.

## Introduction

Multimorbidity, defined as the co-existence of two or more chronic conditions in the same individual [1], has become a dominant global health burden [2, 3]. Prevalence estimates vary widely depending on the definition of multimorbidity, the number and types of conditions included in the definition, and populations studied [4]. A recent study reported between 26.0% to 71.2% in the U.S. depending on the population type (e.g., community-based, institutional) [5]. Governments and health planners have become increasingly aware of this global phenomena, resulting in more requests for population-based research to better conceptualize multimorbidity and understand its determinants and impacts [2]. International research has found multimorbidity to be associated with a range of socio-demographic factors including higher age, female sex, lower education, marital status (divorced, widowed, separated), and rurality [6]. Other studies point to significant negative impacts, linking multimorbidity with greater risk of death, disability, more complex clinical management, reduced self-management ability, and increased healthcare use and costs [3, 7–9]. Prevalence alone does not explain the societal burden of multimorbidity. Studies that have examined disease pairs and clusters suggest that the impacts are complex and variable. For example, Yoon et al. [10] found that the most costly condition clusters had an extremely low prevalence (0.1%-0.4%). Other studies show that the most prevalent conditions/clusters are not necessarily the most burdensome [11–13]. More research is needed to understand which clusters of conditions lead to poor outcomes, and why.

This study examines the association between multimorbidity and burden as measured by functional limitation. Functional limitation is a multidimensional construct that refers to the negative aspects of a person’s ability to live independently and interact with their environment–(i.e., deficits, activity limitations, and restrictions in social participation) [14]. It is often measured by limitations related to: 1) Activities of Daily Living (ADLs), which are basic self-care tasks such as bathing, dressing, eating, and maintaining continence; and/or 2) Instrumental ADLs (IADLs), which are more complex tasks such as preparing meals, housekeeping, and managing finances, medications, transportation and communications [15]. As with multimorbidity, the prevalence of functional limitation varies widely depending on how it is defined, characteristics of the study populations etc. Prevalence estimates of ADL/IADL limitations range from 12% to 54% in community-dwelling older adults [14, 16, 17].

Research suggests that the clustering of mental health disorders with physical comorbidities adds to the complexity of functional limitation. For example, mental health conditions have been shown to affect a range of outcomes including functional limitations and disability in people with multimorbidity, due in part to these conditions being a substantial burden in their own right [18, 19]. Disease clusters that include depressive symptoms were associated with the highest level of disability in the study by Quinones et al. [11] and linked to increased future disability risk in longitudinal research [20]. The negative effects of mental health conditions co-existing with physical comorbidities on IADL limitations and related outcomes have been reported [16]. For example, chronic mental health conditions co-existing with other physical health conditions were associated with significant health-related quality of life (HRQoL) deficits [12, 13], higher health care use and costs [21, 22] and frailty [23]. Yokota et al. [24] examined disability for a large number of individual diseases and disease pairs, and found that depression along with chronic respiratory disease had the highest disability rate and the effect was synergistic (i.e., higher than expected effect based on the addition of the effects of the individual conditions).

Socio-demographic factors also add complexity to studies exploring mental health, multimorbidity and functional limitations (or disability). The prevalence of depression and anxiety in women is almost double that of men [25, 26]. Higher functional limitation is also associated with increasing age, in part due to increasing multimorbidity. However, our work suggests there may be an independent age effect, given that healthcare service use (a consequence of disability) increased with age after controlling for multimorbidity [27]. Studies examining sex differences in functional limitation often show higher rates in women, and attribute this to sex differences in the disablement process and a higher prevalence in women of specific diseases (e.g., depression, arthritis) and disease clusters linked to functionality [28]. The study by Garin et al. [6] and Moore et al’s [29] study on Canadian older adults supports this, showing that women have higher disability and higher rates of arthritis, depression and anxiety.

A number gaps exist in the current research on mental health, multimorbidity and the impacts on functionality. In particular, mental health within the context of multimorbidity is poorly understood, with gaps remaining due to consideration of only a limited set of diseases and the frequent omission of mental health conditions [30–32], separate examination of physical or mental health conditions [33], use of multimorbidity indices that are difficult to translate to clinical practice [34], and concentration on specific age or patient subgroups [2]. A known challenge in capturing mental health conditions has been underdiagnosing and undertreating these conditions in many populations [35, 36]. Garin et al. [6] also highlight the importance of country-specific research, showing both similarities and differences across countries in the risk factors, characteristics and impacts of multimorbidity. In Canada, literature that considers physical and mental comorbidity and its implications is scarce [37]. Mental health, functional limitations, and multimorbidity are priority topics that require continued, frequent research in all countries. Our study responds to this need and general calls for more research on the epidemiology and consequences of multimorbidity [2, 5]. The aim of this study is therefore to provide an understanding of multimorbidity, mental health and impacts on functional limitation in a Canadian, community-dwelling, adult population. To address the under-reporting of mental health conditions, our study includes those with depressive symptoms in addition to those reporting mental health conditions.

## Materials and methods

### Study design and setting

This is a population-based, cross-sectional study that uses data from the Canadian Longitudinal Study on Aging (CLSA), which was launched in 2010 and is one of the largest and most comprehensive research platforms examining health and aging [38]. The CLSA is a 20-year panel study of community-dwelling persons aged 45 to 85 years living in 10 Canadian provinces at recruitment. The CLSA cohort is a nationally stratified sample of 51,338 participants who provided a core set of information on demographics and measures of lifestyle/behavior, social, physical, psychological, and health status. Participants come from one of two CLSA components that differ on sampling design and data collection modes: 1) a ‘tracking’ cohort (n = 21,241) of randomly selected people from the 10 provinces who provided questionnaire data through telephone interviews, and 2) a ‘comprehensive’ cohort (n = 30,097) who were randomly selected from locations within 25–50 km of 11 Data Collection Sites located across Canada who provided data through in-home interviews and site visits. Three main sampling frames were used to recruit into the CLSA cohort: 1) a subset of the Canadian Community Health Survey–Health Aging, which is a nationally representative sample of Canadians > 45 years of age (Tracking only); 2) provincial health registries; and 3) random digit dialing. This study uses baseline CLSA data collected between September 2011 and May 2015. Additional details on the CLSA are provided in (S1 File) and Raina et al. [39].

### Measures

#### Functional limitations

Participants were considered to have a functional limitation if they indicated difficulty with any of 7 ADL or 7 IADL items from the Older Americans Resources and Services (OARS) multidimensional functional assessment [15]. The primary analysis explored functional limitation as a dichotomous variable (any self-reported limitation versus no limitation), and secondary analyses examined dichotomous versions of ADL and IADLs separately to determine whether the overall patterns differed by type of limitation.

#### Multimorbidity

Information on chronic conditions was obtained from self-reported health questions in the CLSA. For each chronic condition, participants were asked “Has a physician ever told you that you have ___?” Participants were asked to report only conditions that lasted, or were expected to last, at least 6 months and were diagnosed by a health professional. Currently, a number of multimorbidity frameworks exist that differ on the chronic conditions included and whether risk factors and symptoms are considered. We selected chronic conditions that were available from the CLSA and included in at least one of the multimorbidity frameworks identified in systematic reviews of the multimorbidity literature and proposed for use by clinicians and researchers [40]. Table 1 provides a list of the chronic conditions obtained from the CLSA data and how they were grouped into 18 categories. The multimorbidity measure used in this study was the number of chronic conditions, with the count created based on the 18 categories (e.g., if a person reported having chronic bronchitis and emphysema, this was counted as one chronic condition since both are included in the Respiratory Conditions group). We operationalized multimorbidity as a count to explore gradient effects, which can otherwise be hidden if multimorbidity is dichotomized using the common threshold of 2 (or 3) conditions [41].

**Table 1:** Summary of chronic conditions grouping for self-reported conditions in the Canadian Longitudinal Study on Aging.

Chronic Condition Category	Specific Conditions Included in Category (where applicable)
1. Arthritis (any)	Osteoarthritis in the knee
	Osteoarthritis in one or both hips
	Osteoarthritis in one or both hands
	Rheumatoid arthritis
2. Mood or Anxiety Disorder	Mood disorder
	Anxiety disorder
3. Respiratory Condition	Asthma
	Emphysema, chronic bronchitis, COPD, or chronic changes in lungs due to smoking
4. Stoke or TIA	Stroke or CVA (cerebrovascular accident)
	Mini-stroke or TIA (Transient Ischemic Attack)
5. Bowel Disorder	Bowel disorder
	Bowel incontinence
6. Eye Condition	Glaucoma
	Cataracts
	Macular degermation
7. Heart Condition	Heart attack or MI
	Heart disease (including CHF)
	Angina (or chest pain due to heart disease)
8. Thyroid Condition	Over-active thyroid gland
	Under-active thyroid gland
9. Neurological Condition	Multiple sclerosis
	Dementia or Alzheimer’s disease
	Epilepsy
	Parkinsonism or Parkinson’s Disease
10. Osteoporosis	
11. High blood pressure or hypertension	
12. Diabetes, borderline diabetes, high blood sugar	
13. Peripheral vascular disease or poor circulation in limbs	
14. Migraine headaches	
15. Intestinal or stomach ulcers	
16. Kidney disease or kidney failure	
17. Urinary incontinence	
18. Cancer (any type)	

#### Mental health conditions

Mental health conditions included self-reported mood and anxiety disorders that had lasted or were expected to last at least 6 months and were diagnosed by a health professional (similar to the other chronic conditions above). For anxiety, participants were asked “Has a doctor ever told you that you have an anxiety disorder such as a phobia, obsessive-compulsive disorder or a panic disorder?” For mood disorders, participants were asked “Has a doctor ever told you that you have a mood disorder such as depression (including manic depression), bipolar disorder, mania, or dysthymia?” The primary analyses used a dichotomous measure comprised of both mood and anxiety disorders (reported a mood or anxiety disorder versus did not report either disorder = ‘with/without mental illness’ in Figs).

Secondary analyses aimed to address the potential under-reporting of mental health disorders by including those with depressive symptoms (in addition to those self-reporting mood and anxiety disorders). This analysis enables exploring symptom burden, which may not be captured in existing diagnoses. Previous studies have shown a positive association between impairments of daily functioning and depression [42] and depressive symptoms [43, 44]. Depressive symptoms have also been shown to be negatively associated with other outcomes such as self-reported health [45, 46] and social support [47]. The 10-item Center for Epidemiological Studies Depression Scale (CESD-10) [48] was used to capture participants with depressive symptoms. The CESD-10 asks participants to rate how often over the past week they experienced symptoms associated with depression, such as restless sleep, poor appetite, and feeling lonely. Response options range from 0 to 3 for each item (0 = Rarely or None of the Time, 1 = Some or Little of the Time, 2 = Moderately or Much of the time, 3 = Most or Almost All the Time). Scores range from 0 to 30, with higher scores indicating greater depressive symptoms. A cut-off of >10 was used, which is recommended based on studies demonstrating acceptable balance across sensitivity, specificity and predictive measures [48, 49]. A score of ≥10 indicates the presence of depressive symptoms severe enough for an individual to be at high risk of depression [50]. Participants with CESD-10 scores ≥ 10 were included in the group having a mental health condition (unless already included due to reporting a mood or anxiety disorder).

#### Socio-demographic variables

In light of the evidence linking socio-demographic factors with mental health, multimorbidity and functional limitations, we included a number of the potential variables that were available in the CLSA, including age, sex, total household income, education level, marital status, living arrangement, and social support. Age ranged from 45 to 86 years; sex was a dichotomous variable (male, female); education level was measured using the categories: no-post-secondary degree, certificate, or diploma”, “education below bachelors’ degree, trade certificate or diploma”, “bachelor’s degree”, and “university degree or certificate above bachelor’s degree”; total household income was measured using the categories: “less than $20,000”, “$20,000 to $49,999”, “$50,000 to $99,999”, $100,000 to $149,999, and “$150,000 or above”; marital status was measured using the categories: “married or living with a partner”, “divorced/separated”, “widowed”, and “never married”; and living arrangement was dichotomous (living with someone, living alone). Social support availability (SSA) was measured by categorizing the continuous score from the 19-item Medical Outcomes Study Social Support Survey [51] into quartiles from 1 (low SSA) through 4 (high SSA).

### Statistical analysis

**Fig 1:**
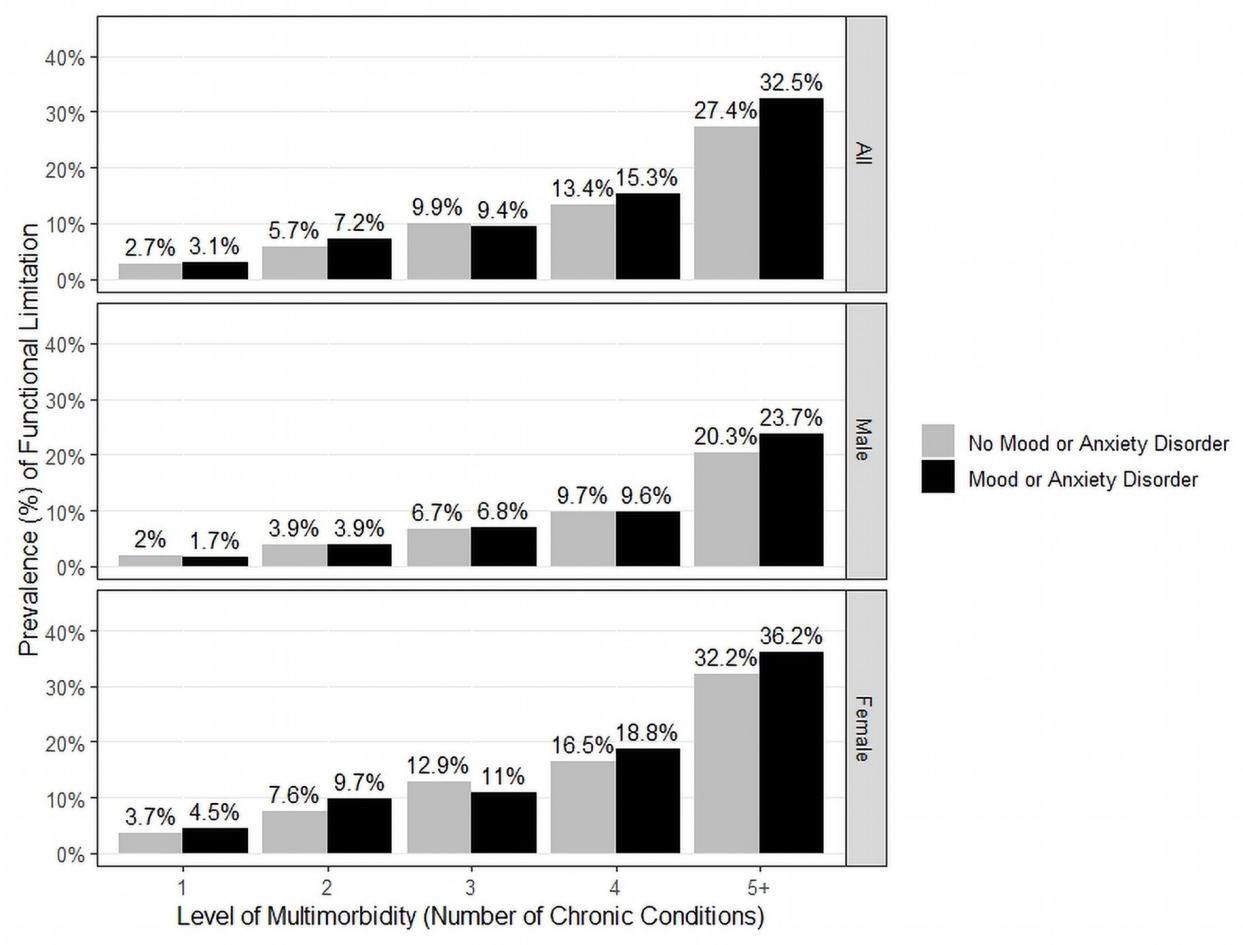
Prevalence of functional limitation by multimorbidity and presence of mood/anxiety disorders (all participants). (A) Prevalence of functional limitation by multimorbidity and presence of mood/anxiety disorders (women). (B) Prevalence of functional limitation by multimorbidity and presence of mood/anxiety disorders (men).

We began by looking at the relationship between the level of multimorbidity and functional limitation. At each level of multimorbidity (see Table 2, Fig 1), we compared the prevalence of functional limitation of those with mental health conditions to those without mental health conditions. Log-linear modelling for a contingency table of frequencies was used to assess the statistical significance of the associations between these three discrete variables (level of multimorbidity [1–5+], presence of mental health conditions [Yes/No], functional limitation [Yes/No]) [52]. We then further stratified this analysis by each of the socio-demographic variables to determine whether these variables were potential confounders or effect modifiers. Our aim was to identify key variables to reduce the socio-demographic covariates in the regression models thereby avoiding overparameterization (due to the small number of CLSA participants having multimorbidity and/or functional limitations). Log-linear modelling combined with visual inspection of the stratified plots was used to select the subset of socio-demographic variables representing the key covariates. Logistic regression was used to examine the relationship between functional limitation (dependent variable, dichotomous, composite of ADL/IADL limitations) and level of multimorbidity (independent variable), with key potential covariates in all models. Separate models were run for multimorbidity that did and did not include mental health conditions, and the odds ratios for each multimorbidity level were compared (e.g., for a multimorbidity level of two, those with two physical chronic conditions were compared to those with one physical and one mental health condition). Tests of interaction were conducted to compare odds ratios at each level of multimorbidity [53]. A reference of 0 for the level of multimorbidity was used in the models. Sensitivity analyses were run to determine if the results differed by the type of functional limitation (ADL vs IADL) and if mental health conditions included those with depressive symptoms (in addition to those reporting mood or anxiety disorders).

**Table 2:** Characteristics of the 51,338 participants of the Canadian Longitudinal Study on Aging.

Characteristic	All Participants (n = 51,338)		Participants with Mood or Anxiety Disorders (n = 10,070)		Participants without Mood or Anxiety Disorders (n = 41,113)		Significance Test	
	N	%	N	%	N	%	Χ^2^	p-value
Sex								
Men	25183	49.1	3731	37.1	21370	52.0	726.3	<0.0001
Women	26155	51.0	6339	62.9	19743	48.0		
Age								
45–54	13427	26.2	2961	29.4	10430	25.4	508.1	<0.0001
55–64	16420	32.0	3749	37.2	12625	30.7		
65–74	11996	23.4	2213	22.0	9747	23.7		
75+	9495	18.5	1147	11.4	8311	20.2		
5 Most Common Chronic Conditions								
Arthritis (osteoarthritis, rheumatoid arthritis)	14825	29.3	3514	34.9	6423	15.6	1920.4	<0.0001
Eye condition (cataracts, glaucoma, macular degeneration)	15608	30.9	3026	30.0	6875	16.7	920.8	<0.0001
Hypertension	19203	37.6	4033	40.0	6012	14.6	3315.3	<0.0001
Diabetes, borderline diabetes, or high blood sugar	8863	17.3	2137	21.2	7918	19.3	19.7	<0.0001
Respiratory condition (emphysema, chronic bronchitis, chronic obstructive pulmonary disease, chronic changes in lungs due to smoking)	8379	16.4	2376	23.6	7645	18.6	128.4	<0.0001
Number of Chronic Conditions (excluding mood or anxiety conditions)								
0	7564	14.8	0	0.0	7564	100.0	5329.7	<0.0001
1	10532	20.6	1103	10.5	9429	89.5		
2	10339	20.2	1826	17.7	8513	82.3		
3	8248	16.1	1922	23.3	6326	76.7		
4	5875	11.5	1660	28.3	4215	71.7		
5+	8625	16.9	3559	41.3	5066	58.7		
Social Participation Prevented by Health Status	3877	7.6	1424	14.1	2438	5.9	781.7	<0.0001
ADL/IADL Limitation	5186	10.1	1732	17.2	3425	8.3	702.2	<0.0001

Exploratory factor analysis was used to identify the other conditions that frequently cluster with mental health disorders. Factor analysis highlights clusters of statistically significant co-occurrences of chronic conditions, which sheds light on common disease patterns, the overlap in diagnostic groups, and the potential for co-occurring conditions to influence each other and health outcomes such as functional limitation [54]. This was done to better understand the associations with disability and potential for synergistic effects arising from combinations of mental and physical health conditions. We used the principal factor method, a tetrachoric correlation matrix (due to the dichotomous nature of the chronic conditions data), and a varimax rotation to aid in the interpretation of the factors as multimorbidity patterns reflecting clusters of diagnosis groups [54]. A condition was defined to be associated with a factor (cluster) if it had a factor loading from the varimax rotation of 0.40 or more. There is much debate about choosing a factor loading threshold for assigning variables to factors; however, values within the range of 0.40–0.50 are common [55] and align with those used in multimorbidity research [54]. Other factors are also important in assigning items to factors, particularly the conceptual/empirical foundation underlying the work. The scree plot and Kaiser criterion (eigenvalues greater than one) were used to determine the number of factors to extract from the model.

SAS version 9.4 was used for all statistical analyses, and a 95% confidence level (5% alpha) was assumed.

### Ethics

This research was made possible through the data collected by the Canadian Longitudinal Study on Aging (CLSA). Funding for the CLSA is provided through the Government of Canada through the Canadian Institutes of Health Research under grant reference LSA9447 and the Canada Foundation for Innovation. CLSA is the authorized data custodian and obtained written consent from all participants. This study, a secondary use of CLSA data, was approved by the Hamilton Integrated Research Ethics Board (Ethics Certificate No. 3387-C pertaining to an application for “Retrospective Review of Medical Charts/Health Records”). All data used in this study were fully anonymized before they were accessed by the research team.

## Results

Table 2 displays selected characteristics of the CLSA participants. Overall, the proportion of men and women was similar (49% versus 51%), with about one quarter (26%) between 45 and 54 years of age and 42% that were 65 years of age or older. Fifteen percent (15%) of participants had no chronic conditions and 65% had two or more (multimorbidity). The mean number of chronic conditions was 2.23, with the most common chronic conditions being hypertension (37%), eye conditions and arthritis (about 30% each), diabetes (17%) and respiratory conditions (16%). Approximately 8% of participants said that their health prevented them from participating socially, and 10% of participants reported at least one ADL or IADL limitation.

Table 2 also highlights key differences in those with and without a mood or anxiety disorder, all of which were statistically significant. Mood and anxiety disorders were reported by 10,070 (20%) of the participants, and were more prevalent in younger age groups (45–54 and 55–64) and women. Multimorbidity was also more common in those with a mood/anxiety disorder versus without a mood/anxiety disorder, with the mean number of chronic conditions being 2.82 in those with mood/anxiety versus 2.09 without. This is notable, given that multimorbidity normally increases with age, yet mental health conditions are more prevalent in the younger age groups. Social participation restrictions due to health were more frequently cited by those with mood/anxiety (14.1%) versus without (5.9%), as were functional limitations (ADL/IADL) (17.2% & 8.3% for those with versus without mood/anxiety disorders).

Fig 1 shows the prevalence of functional limitation by multimorbidity for all CLSA participants. The proportion of participants reporting functional limitations increases consistently with the level of multimorbidity, from 3% for those with one chronic condition to 32% for those with 5+ conditions. Fig 1A and 1B show that the same trend is seen in both women and men (respectively), with the proportion reporting functional limitation being higher in women compared to men at every level of multimorbidity. There is also evidence that for both sexes those with mood/anxiety disorders report a higher prevalence of functional limitation compared to those without (e.g., for 5+ chronic conditions, the proportion of women with functional limitation in those with vs without mood/anxiety disorders is 37% vs. 32%, and for men is 23% vs 20%). Log-linear model results for *all CLSA* participants and for *women only* show statistically-significant associations between all three variables for the best-fitting model, a model equivalent to a logistic regression model that includes level of multimorbidity and mood/anxiety disorders as independent predictors of functional limitation with no interaction term (see S2 and S3 Files for detailed statistical results for all CLSA participants and women only, respectively). Log-linear model results for *men* show that level of multimorbidity but not mood is statistically significantly associated with functional limitation (see S4 File).

**Fig 2:**
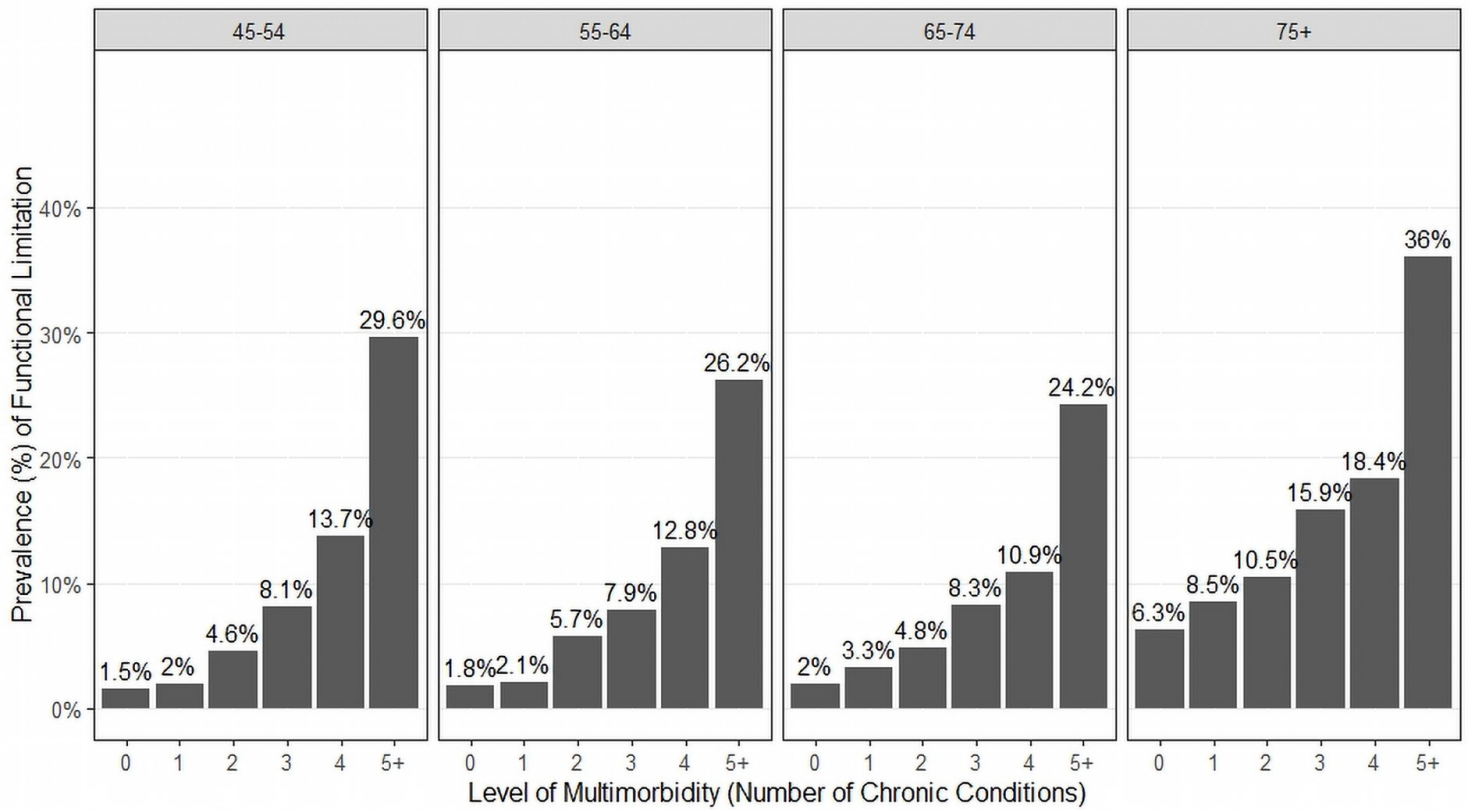
Prevalence of functional limitation by multimorbidity and age.

One of the strongest relationships with multimorbidity is seen with age, therefore we conducted a stratified analysis to explore the independent effects of age and multimorbidity on functional limitation. Fig 2 shows that the proportion with functional limitation increased steadily with the level of multimorbidity in all age groups. The proportion with functional limitation for a given level of multimorbidity often increased with age, but the age effect was less pronounced and consistent compared to the multimorbidity effect. Log-linear model results for the variables in Fig 2 show statistically-significant associations between all three variables for the best-fitting model, a model equivalent to a logistic regression model that includes level of multimorbidity, age and the interaction term (level of multimorbidity x age) as predictors of functional limitation (see S5 File). Log-linear modelling was also conducted to explore associations between functional limitation and factors other than age, including sex, total household income, education level, living arrangement (and marital status), and social support. All log-linear models showed statistically-significant associations between each of these variables and functional limitation, after controlling for multimorbidity (see S6 File). Given that statistical significance was driven by the large sample, further examination of the stratified plots was done to identify the variables showing the strongest association with functional limitation. These analyses suggested that age, sex, and education showed the strongest relationships with functional limitation (e.g., more functional limitation with older age, female sex, lower education), thus, all logistic regressions were adjusted for these variables.

**Fig 3:**
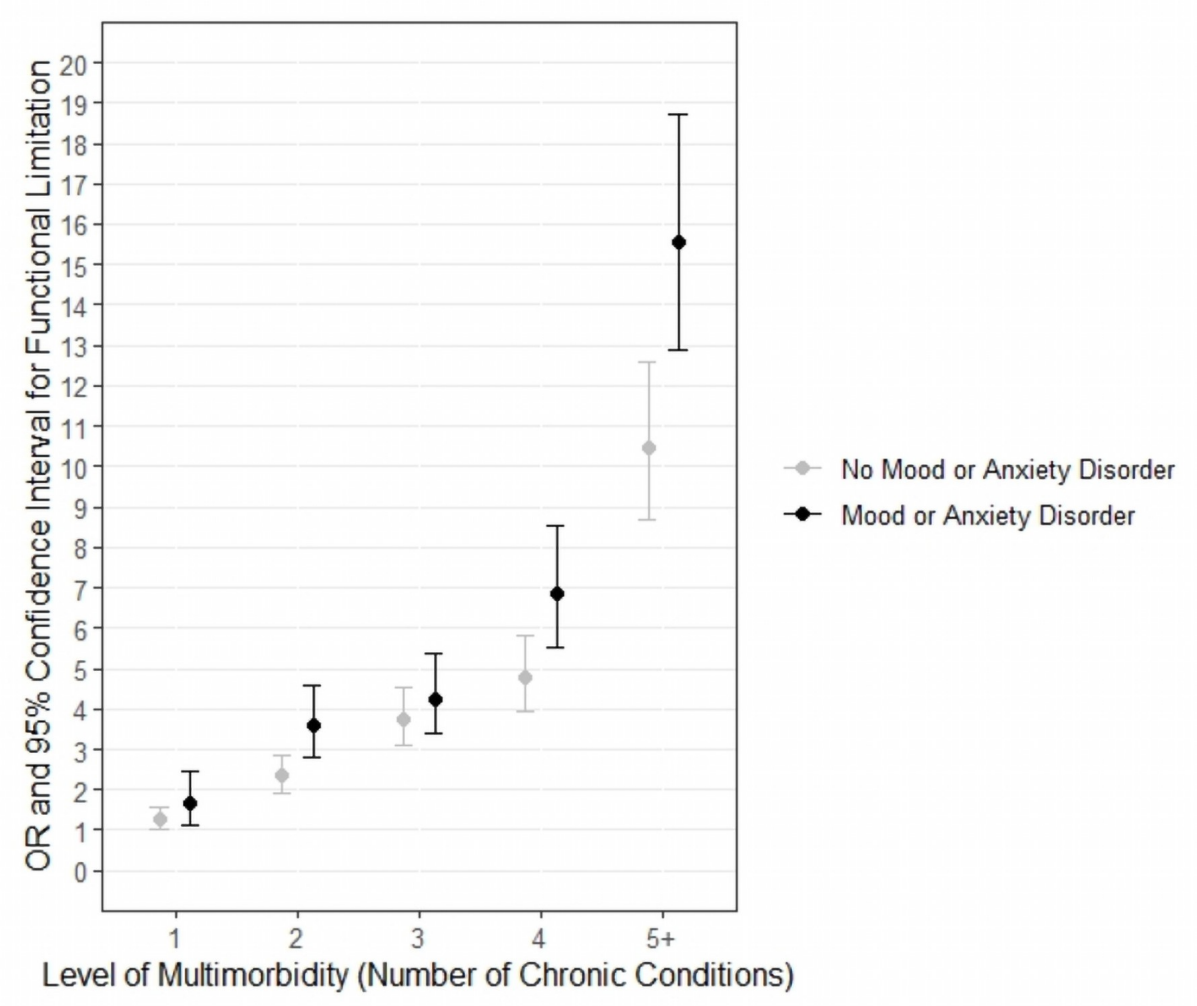
Odds of functional limitation by multimorbidity and presence of mood/anxiety disorders (reference category—0 chronic conditions, models adjusted for age, sex, education).

Fig 3 shows that the odds of having a functional limitation consistently increased with the level of multimorbidity. The point estimate of the odds of functional limitation from 1 to 5+ chronic conditions increased from 1.9 to 15.8 in those with mood/anxiety disorders versus 1.8 to 10.2 for those without mood/anxiety disorders. For three of the five multimorbidity levels (2, 4, 5+), significantly higher ORs were seen in those having mood/anxiety disorders compared to those without (Level 2: z = 2.68, p = 0.007; Level 4: z = 2.42, p = 0.016; Level 5+: z = 2.94, p = 0.003). This figure also suggests that it is important to consider multimorbidity when examining mood disorders and functional limitation, since a range of ORs exist for those with mood disorders depending on multimorbidity and differences can be significant, especially at high multimorbidity levels (e.g., OR confidence intervals overlap for ‘Mood 2’and ‘Mood 3”, but not for ‘Mood 3’ and ‘Mood 4’ or ‘Mood 4’ and ‘Mood 5+’.

**Fig 4:**
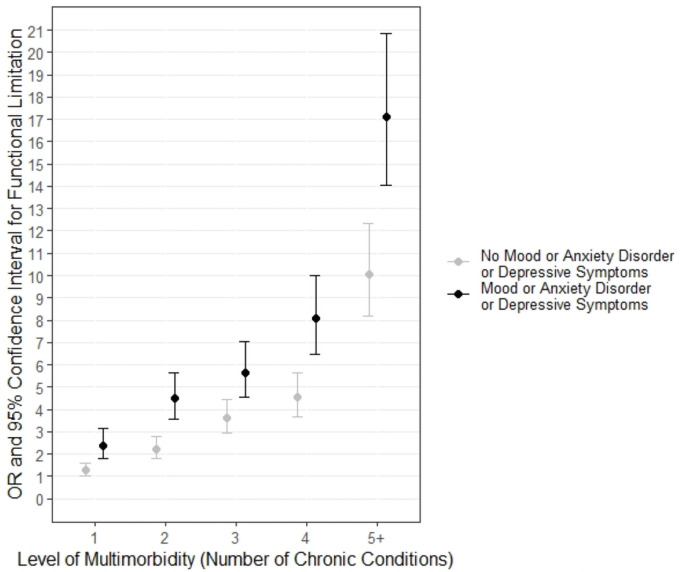
Odds of functional limitation by multimorbidity and presence of mood/anxiety/CESD disorders (reference category—0 chronic conditions, models adjusted for age, sex, education).

Fig 4 shows the results of the regressions run using the composite mental health measure (combined self-reported mood/anxiety + CESD score 10+). A total of 3,159 participants had a CESD score 10+, with 1,527 already captured in those with self-reported mood/anxiety (i.e., 1,632 additional participants were captured by the composite measure). The odds of functional limitation consistently increased with the level of multimorbidity as we saw in Fig 3, but now the point estimate ORs were significantly higher for those with mental health disorders compared to those without for all levels of multimorbidity (Level 1: z = 3.30, p = 0.001; Level 2: z = 4.36, p<0.0001; Level 3: z = 2.88, p = 0.004; Level 4: z = 3.65, p = 0.0003; Level 5+: z = 3.66, p = 0.0003). As with Fig 3, we see a range of ORs for those with mood disorders depending on the level of multimorbidity, with larger differences at only certain levels of multimorbidity (e.g., ‘Mood 1’ and ‘Mood 2’, ‘Mood 4’ and ‘Mood 5+”). Separate regressions were also run with functional limitation defined by the two individual types (ADL, IADL). Patterns similar to Fig 3 were seen for both ADLs and IADLs, although the ORs were higher with more overlap in the confidence intervals for IADLs compared to ADLs (see S1 and S2 Figs).

**Fig 5:**
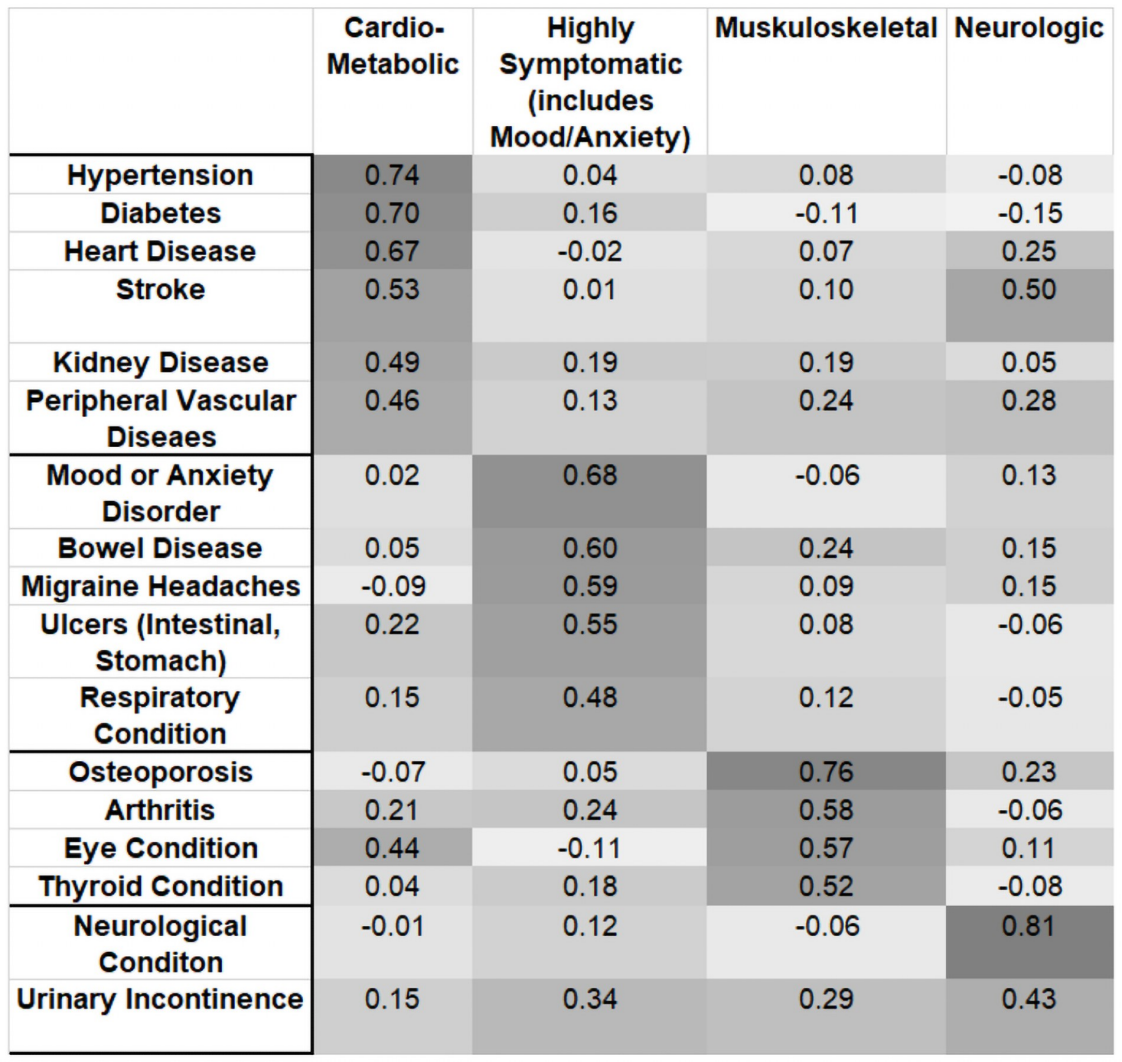
Chronic condition clusters—factor loadings from rotated factor analysis.

Fig 5 shows the factor loadings from the four factor (condition cluster) model that resulted from the rotated factor analysis. Conditions included in each factor are indicated by the darker grey shading, with a threshold of 0.40 or more used to determine the association with a factor. Applying this threshold generally resulted in a clear assignment of conditions to factors with few cross-loading concerns. The mood/anxiety cluster also included bowel and gastrointestinal disorders, migraine headaches, and respiratory conditions. The other condition clusters included a cardio-metabolic cluster containing a number of cardio-vascular conditions and diabetes; a neurologic cluster containing Parkinson’s disease, various dementias, and urinary incontinence; and musculoskeletal cluster containing osteoporosis, arthritis, thyroid conditions, and various eye conditions. The same four factors and similar factor loadings were observed when the model was re-run to include those with depressive symptoms (CESD-10 score 10+) in the group with mental health disorders (data not shown).

## Discussion

This study makes an important contribution to the literature by considering mental health conditions in the measure of multimorbidity and exploring the disproportionate impact these may have on functional limitations when present with other physical conditions. This study highlighted the stronger association of comorbid mental and physical conditions compared to physical conditions alone with ADL/IADL functioning in an adult, community-dwelling population. Functional limitation, defined in this study as those with at least one ADL or IADL limitation, was 10% overall in our cohort. This is broadly consistent with proportions reported in the literature, given that our study included adults aged 45+ years and the range in the literature is 12% to 54% for community-dwelling older adults [14, 16]. Higher functional limitation and multimorbidity in those with mental health disorders has also been reported in the recent literature. For example, Schousboe et al. [19] found that for a Geriatric Depression Score of 0–1 versus 6+, the proportion with 4+ medical conditions increased from 16% to 33% and the proportion with IADL impairments rose from 14% to 58%. Similarly, a meta-analysis of population data involving 190,593 people across 43 low and middle-income countries reported odds of multimorbidity of 2.62 versus 3.44 for those with no depression versus those with depressive episodes [56].

Functional limitation is positively correlated with both age and multimorbidity, thus we controlled for age (as well as sex and education) in examining the associations of multimorbidity and functional limitation. In the adjusted models, we found that the odds of functional limitation rose steadily with increasing multimorbidity and were higher for those with versus without mental health disorders. Two recent Canadian studies provide supportive evidence of the negative effects of mental and physical co-morbidity: Guerra et al. [16] reported higher odds of IADL disability for multimorbidity clusters that included mental health conditions compared to those without in a study of older adults from Quebec (Canada), and Dai et al.’s study of a nationally-representative population [37] found that in people with the same number of chronic conditions those with mental disorders or physical and mental comorbidity showed higher prevalence of functional limitations (impaired HRQoL, higher service use). Studies from other countries show similar effects for functional limitations [57] and related outcomes such as frailty [23], HRQoL [12, 13, 57], subjective well-being [33], and service use/cost [19].

The adverse impact of co-existing mental and physical chronic conditions on functional limitation has significant implications for policy, clinical practice and research. Early screening and intervention to address mental disorders is recommended in best practice guidelines for multimorbidity and should be given priority to prevent the onset and progression of functional limitation. This is particularly important in older adults, where multimorbidity and mental health disorders such as anxiety and depression are both highly prevalent [2, 58]. Mental health conditions are also more common among those with physical health conditions [16, 59], which may reflect a dose-response or bidirectional relationship between mood/anxiety disorders and physical health conditions [16, 60–62]. Our finding that multimorbidity was higher in those with versus without a mental health condition despite mental health conditions being more common in the younger age groups highlights the potentially-powerful influence of mental health conditions. There is considerable evidence in the literature that multimorbidity increases with age [4], yet in our study multimorbidity was higher in those with mental health conditions, which were more common in younger age groups. This is consistent with evidence that the impact of multimorbidity in younger ages can be more significant than in older ages, perhaps because younger people with serious health conditions face multiple vulnerabilities. This may also reflect negative perceptions among younger people with multimorbidity who compare themselves to peers from the same generation where multimorbidity is uncommon.

Unfortunately, mental health conditions remain underdiagnosed and undertreated in the general population [35] and in people from low and middle-high income countries alike [36], perhaps due to stigma, discrimination or access barriers faced by people with mental health conditions [63]. Underdiagnosing and undertreating mental health disorders can result in negative feedback cycles that reinforce the progression of chronic illness and functional limitation. It also impacts research–e.g., administrative and self-report data sources will not be inclusive of all those with mental health disorders, thus the full impact of mental health on the outcomes estimated from studies relying on these sources is likely to be underestimated. A unique aspect of our study was addressing the under-reporting of mental health conditions by including those with depressive symptoms (CESD-10 score of 10+) in addition to those reporting mental health conditions. The inclusion of these participants may be why we observed higher odds of functional limitation in the group with mental health disorders (compared to the odds when these participants were not included in the group). This may highlight an important difference between symptoms and diagnoses. People may live with a variety of symptoms of either depression or anxiety that impact their functional ability, yet never receive a diagnosis of mental illness for various reasons.

Factor analysis highlights the conditions mental health disorders cluster with, which can shed light on potential clinical complexities and impacts on functional limitation. Despite heterogeneity in the multimorbidity clusters identified in the existing literature, the systematic review by Prados-Torres et al. [64] shows considerable support for the clustering of cardio-metabolic, musculoskeletal (e.g., arthritis, osteoporosis), and mental health conditions. Our main interest was the mental health cluster, and here we find several other studies supporting our findings that link mental health problems with bowel and gastrointestinal disorders [54, 65, 66], respiratory conditions [24, 54, 67, 68], and migraine headaches [54, 69, 70]. Our results are also consistent with studies linking mood/anxiety disorders with ‘somatic symptoms’, which are those that defy a clear organic/medical explanation and include the gastrointestinal symptoms and migraine headaches that appeared in our mental health cluster [69, 71]. This link has been seen often enough that the term ‘cosyndromality’ has been suggested for the concurrent occurrence of somatic, anxiety and depressive symptoms [72]. This clustering of conditions has at least two important implications in relation to our study. First, it may partially explain the underdiagnosing and undertreating of mental health conditions. Somatic conditions defy an organic explanation and as such may be undertreated, and while these conditions may trigger a physician/clinic visit there is little reason to expect discordant conditions to be further investigated. This situation contrasts with the cardio-metabolic cluster, where the association between cardiovascular and metabolic disorders has been long known [54], thus the cluster of conditions is anticipated with concurrent diagnosis and treatment of the suite of conditions likely to be pursued. In general, mental illness that accompanies physical conditions is less well detected than mental illness alone, even when it is known that physical illness causes mental illness [59]. Clearly, there are improvements needed in detecting and treating mental illness accompanying physical disorders. Second, both somatic and mental health conditions likely shape functional limitation. There is evidence cross-sectionally and longitudinally that somatization contributes to the presence of functional limitation, an effect that remains even after anxiety and depressive disorders are taken into account [73, 74]. At the higher levels of multimorbidity in our study (i.e., 2+), the higher odds of functional limitation we found in those with versus without mental health conditions may be due to mood/anxiety disorders, the somatic conditions they often cluster with, or interactions among these conditions. More research is needed to understand what the combination of conditions in the mental health cluster means for functional limitation. There is some evidence that chronic respiratory conditions co-occurring with depression are linked to higher disability [24]. There is also research showing that depression and somatic symptoms are independently associated with disability; however, disentangling the effects and identifying multiplicative effects has proven challenging [75]. Our factor analysis results support the existence of multiplicative effects, but further evidence is needed to strengthen this claim (e.g., comparison of conditions and associated disability in those with and without mental health disorders at each multimorbidity level).

### Limitations

We acknowledge several limitations related to our study. Our measure of multimorbidity included self-reported chronic conditions available from the CLSA dataset. These conditions represent the most prevalent chronic conditions in Canadian older adults, those included in widely-recognized multimorbidity frameworks, and those recommended for use in multimorbidity research [76]. However, their self-reported nature, the conditions included and grouping of conditions, and the existence of conditions not captured by CLSA can change the measure of multimorbidity or mental health, and thus the analyses that use these measures (e.g., relationship with functional limitations, factor analyses). Griffith et al. note that inclusion of conditions regarded as symptoms (e.g., bowel disorders, migraine headaches, urinary incontinence) increases multimorbidity prevalence and associations of multimorbidity with outcomes such as functional limitation. Also, our measure of multimorbidity—the number of chronic conditions—does not capture differences in the types, combinations or severity of the conditions, all of which impact functional limitation. Essentially, we still lack a consistent definition or framework for thinking about multimorbidity, which impacts research and the comparability of studies [77]. We also did not compare the conditions at each level of multimorbidity for those with and without mental health disorders, which would help to strengthen conclusions relating to the impacts of specific condition clusters. Our results are also influenced by the underdetection and undertreatment of mental health disorders. We attempted to address this by including those with depressive symptoms in the group with mental health disorders, but this approach would not be as strong as relying on diagnostic information. While higher false positive rates (and related impacts to resources/patients) is a consequence of follow up on screening tools such as the CESD-10, perhaps this is tolerable when balanced against the risks arising from underdetection [49]. It should also be acknowledged that CLSA participants are relatively healthy with low levels of multimorbidity and particularly functional limitation, thereby limiting our ability to conduct stratified analyses, explore gradient effects for functional limitation and limiting the generalizability of our findings to the general community-dwelling adult population. Finally, this study used available baseline CLSA data, which is cross-sectional. As such, causal claims cannot be made regarding the impact of mental health disorders on functional limitation in people with multimorbidity, nor can we rule out bi-directional effects of functional limitation on multimorbidity and mental health [42]. While evidence continues to accumulate regarding the negative effects of mental health conditions co-existing with physical health conditions, longitudinal analyses are required to better understand reciprocal relationships and the impacts on functional limitation and related outcomes.

## Conclusions

This study found higher odds of having a functional limitation for those with versus without mental health conditions, at all levels of multimorbidity. Larger differences were seen when mental health conditions were broadened beyond diagnoses to include depressive symptoms, which may reflect underdetection and undertreatment of mental illness. These results generally support the need for concurrent and proactive identification and management of mental and physical comorbidities in order to prevent functional limitation, future decline, and the associated burdens identified in the literature (e.g., reduced quality of life, healthcare & service costs). Primary and secondary interventions should target people with mood/anxiety conditions coexistent with physical health conditions, consistent with the alerts to common mental health disorders (anxiety, depression) highlighted in the NICE clinical assessment and management guidelines for people with multimorbidity (https://www.nice.org.uk/guidance/qs153).

## Supporting information

S1 Fig Odds of ADL limitation by multimorbidity and presence of mood/anxiety/CESD disorders (reference category—0 chronic conditions, models adjusted for age, sex, education). (JPEG)

S2 Fig Odds of IADL limitation by multimorbidity and presence of mood/anxiety/CESD Disorders (reference category—0 chronic conditions, models adjusted for age, sex, education). (JPEG)

S1 File CLSA summary. Additional information about the Canadian Longitudinal Study on Aging.
(DOCX)

S2 File Loglinear model results for Fig 1 (all participants). (DOCX)

S3 File Loglinear model results for Fig 1A (women). (DOCX)

S4 File Loglinear model results for Fig 1B (men). (DOCX)

S5 File Loglinear model results for Fig 2 (age). (DOCX)

S6 File Loglinear model results for sociodemographic variables. (DOCX)

## References

[1] Smith S.M., et al., Interventions for improving outcomes in patients with multimorbidity in primary care and community settings. Cochrane Database Syst Rev, 2016. 3: p. CD006560. 26976529 10.1002/14651858.CD006560.pub3 PMC6703144

[2] Marengoni A., et al., Aging with multimorbidity: a systematic review of the literature. Ageing Res Rev, 2011. 10(4): p. 430–9. doi: 10.1016/j.arr.2011.03.003 21402176

[3] Tinetti M.E., Fried T.R., and Boyd C.M., Designing health care for the most common chronic condition—multimorbidity. JAMA, 2012. 307(23): p. 2493–4. doi: 10.1001/jama.2012.5265 22797447 PMC4083627

[4] Hernandez B., Reilly R.B., and Kenny R.A., Investigation of multimorbidity and prevalent disease combinations in older Irish adults using network analysis and association rules. Sci Rep, 2019. 9(1): p. 14567. doi: 10.1038/s41598-019-51135-7 31601959 PMC6787335

[5] Goodman R.A., et al., Multimorbidity Patterns in the United States: Implications for Research and Clinical Practice. J Gerontol A Biol Sci Med Sci, 2016. 71(2): p. 215–20. doi: 10.1093/gerona/glv199 26714567

[6] Garin N., et al., Global Multimorbidity Patterns: A Cross-Sectional, Population-Based, Multi-Country Study. J Gerontol A Biol Sci Med Sci, 2016. 71(2): p. 205–14. doi: 10.1093/gerona/glv128 26419978 PMC5864156

[7] Salive M.E., Multimorbidity in older adults. Epidemiol Rev, 2013. 35: p. 75–83. doi: 10.1093/epirev/mxs009 23372025

[8] Lehnert T., et al., Review: health care utilization and costs of elderly persons with multiple chronic conditions. Med Care Res Rev, 2011. 68(4): p. 387–420. doi: 10.1177/1077558711399580 21813576

[9] Violan C., et al., Burden of multimorbidity, socioeconomic status and use of health services across stages of life in urban areas: a cross-sectional study. BMC Public Health, 2014. 14: p. 530. doi: 10.1186/1471-2458-14-530 24885174 PMC4060853

[10] Yoon J., et al., Costs associated with multimorbidity among VA patients. Med Care, 2014. 52 Suppl 3: p. S31–6. doi: 10.1097/MLR.0000000000000061 24561756 PMC8051430

[11] Quinones A.R., et al., Prospective Disability in Different Combinations of Somatic and Mental Multimorbidity. J Gerontol A Biol Sci Med Sci, 2018. 73(2): p. 204–210. doi: 10.1093/gerona/glx100 28541396 PMC6279134

[12] Mujica-Mota R.E., et al., Common patterns of morbidity and multi-morbidity and their impact on health-related quality of life: evidence from a national survey. Qual Life Res, 2015. 24(4): p. 909–18. doi: 10.1007/s11136-014-0820-7 25344816 PMC4366552

[13] Brettschneider C., et al., Relative impact of multimorbid chronic conditions on health-related quality of life—results from the MultiCare Cohort Study. PLoS One, 2013. 8(6): p. e66742. doi: 10.1371/journal.pone.0066742 23826124 PMC3691259

[14] Millan-Calenti J.C., et al., Prevalence of functional disability in activities of daily living (ADL), instrumental activities of daily living (IADL) and associated factors, as predictors of morbidity and mortality. Arch Gerontol Geriatr, 2010. 50(3): p. 306–10. doi: 10.1016/j.archger.2009.04.017 19520442

[15] Fillenbaum G.G. and Smyer M.A., The development, validity, and reliability of the OARS multidimensional functional assessment questionnaire. J Gerontol, 1981. 36(4): p. 428–34. doi: 10.1093/geronj/36.4.428 7252074

[16] Gontijo Guerra S., Berbiche D., and Vasiliadis H.M., Changes in instrumental activities of daily living functioning associated with concurrent common mental disorders and physical multimorbidity in older adults. Disabil Rehabil, 2020: p. 1–9. doi: 10.1080/09638288.2020.1745303 32255362

[17] St John P.D., et al., Multimorbidity, disability, and mortality in community-dwelling older adults. Can Fam Physician, 2014. 60(5): p. e272–80. 24829022 PMC4020665

[18] Cassano P. and Fava M., Depression and public health: an overview. J Psychosom Res, 2002. 53(4): p. 849–57. doi: 10.1016/s0022-3999(02)00304-5 12377293

[19] Schousboe J.T., et al., Depressive Symptoms and Total Healthcare Costs: Roles of Functional Limitations and Multimorbidity. J Am Geriatr Soc, 2019. 67(8): p. 1596–1603. doi: 10.1111/jgs.15881 30903701 PMC6684454

[20] Murphy R.A., et al., Depressive Trajectories and Risk of Disability and Mortality in Older Adults: Longitudinal Findings From the Health, Aging, and Body Composition Study. J Gerontol A Biol Sci Med Sci, 2016. 71(2): p. 228–35. doi: 10.1093/gerona/glv139 26273025 PMC4723662

[21] Bock J.O., et al., Impact of depression on health care utilization and costs among multimorbid patients—from the MultiCare Cohort Study. PLoS One, 2014. 9(3): p. e91973. doi: 10.1371/journal.pone.0091973 24638040 PMC3956806

[22] Luppa M., et al., Direct costs associated with depressive symptoms in late life: a 4.5-year prospective study. Int Psychogeriatr, 2013. 25(2): p. 292–302. doi: 10.1017/S1041610212001688 23083505

[23] Bekic S., et al., Clustering of Mental and Physical Comorbidity and the Risk of Frailty in Patients Aged 60 Years or More in Primary Care. Med Sci Monit, 2019. 25: p. 6820–6835. doi: 10.12659/MSM.915063 31507272 PMC6753844

[24] Yokota R.T., et al., Impact of Chronic Conditions and Multimorbidity on the Disability Burden in the Older Population in Belgium. J Gerontol A Biol Sci Med Sci, 2016. 71(7): p. 903–9. doi: 10.1093/gerona/glv234 26774118

[25] Agur K., et al., How Does Sex Influence Multimorbidity? Secondary Analysis of a Large Nationally Representative Dataset. Int J Environ Res Public Health, 2016. 13(4): p. 391. doi: 10.3390/ijerph13040391 27043599 PMC4847053

[26] Faravelli C., et al., Gender differences in depression and anxiety: the role of age. Psychiatry Res, 2013. 210(3): p. 1301–3. doi: 10.1016/j.psychres.2013.09.027 24135551

[27] Fisher K., et al., Comorbidity and its relationship with health service use and cost in community-living older adults with diabetes: A population-based study in Ontario, Canada. Diabetes Res Clin Pract, 2016. 122: p. 113–123. doi: 10.1016/j.diabres.2016.10.009 27833049

[28] Arbeev K.G., et al., Disability trends in gender and race groups of early retirement ages in the USA. Soz Praventivmed, 2004. 49(2): p. 142–51. doi: 10.1007/s00038-004-3041-y 15150866

[29] Moore E.G., Rosenberg M.W., and Fitzgibbon S.H., Activity limitation and chronic conditions in Canada’s elderly, 1986–2011. Disabil Rehabil, 1999. 21(5–6): p. 196–210. doi: 10.1080/096382899297620 10381232

[30] Stenholm S., et al., Age-related trajectories of physical functioning in work and retirement: the role of sociodemographic factors, lifestyle and disease. J Epidemiol Community Health, 2014. 68(6): p. 503–9. doi: 10.1136/jech-2013-203555 24534071

[31] Loza E., et al., Multimorbidity: prevalence, effect on quality of life and daily functioning, and variation of this effect when one condition is a rheumatic disease. Semin Arthritis Rheum, 2009. 38(4): p. 312–9. doi: 10.1016/j.semarthrit.2008.01.004 18336872

[32] Guralnik J.M., et al., Maintaining mobility in late life. I. Demographic characteristics and chronic conditions. Am J Epidemiol, 1993. 137(8): p. 845–57. doi: 10.1093/oxfordjournals.aje.a116746 8484376

[33] Wang S.Y. and Kim G., The Relationship between Physical-Mental Comorbidity and Subjective Well-Being among Older Adults. Clin Gerontol, 2020. 43(4): p. 455–464. doi: 10.1080/07317115.2019.1580810 30831062

[34] Diederichs C., Berger K., and Bartels D.B., The measurement of multiple chronic diseases—a systematic review on existing multimorbidity indices. J Gerontol A Biol Sci Med Sci, 2011. 66(3): p. 301–11. doi: 10.1093/gerona/glq208 21112963

[35] Gonzalez H.M., et al., Depression care in the United States: too little for too few. Arch Gen Psychiatry, 2010. 67(1): p. 37–46. doi: 10.1001/archgenpsychiatry.2009.168 20048221 PMC2887749

[36] Ormel J., et al., Disability and treatment of specific mental and physical disorders across the world. Br J Psychiatry, 2008. 192(5): p. 368–75. doi: 10.1192/bjp.bp.107.039107 18450663 PMC2681238

[37] Dai H., et al., Epidemiology of physical and mental comorbidity in Canada and implications for health-related quality of life, suicidal ideation, and healthcare utilization: A nationwide cross-sectional study. J Affect Disord, 2020. 263: p. 209–215. doi: 10.1016/j.jad.2019.11.146 31818778

[38] Raina P.S., et al., The Canadian longitudinal study on aging (CLSA). Can J Aging, 2009. 28(3): p. 221–9. doi: 10.1017/S0714980809990055 19860977

[39] Raina P., et al., Cohort Profile: The Canadian Longitudinal Study on Aging (CLSA). Int J Epidemiol, 2019. 48(6): p. 1752–1753j. doi: 10.1093/ije/dyz173 31633757 PMC6929533

[40] Griffith L.E., et al., Multimorbidity Frameworks Impact Prevalence and Relationships with Patient-Important Outcomes. J Am Geriatr Soc, 2019. 67(8): p. 1632–1640. doi: 10.1111/jgs.15921 30957230

[41] Willadsen T.G., et al., The role of diseases, risk factors and symptoms in the definition of multimorbidity—a systematic review. Scand J Prim Health Care, 2016. 34(2): p. 112–21. doi: 10.3109/02813432.2016.1153242 26954365 PMC4977932

[42] Karp J.F., et al., Use of the late-life function and disability instrument to assess disability in major depression. J Am Geriatr Soc, 2009. 57(9): p. 1612–9. doi: 10.1111/j.1532-5415.2009.02398.x 19682111 PMC2854008

[43] Bruce M.L., et al., The impact of depressive symptomatology on physical disability: MacArthur Studies of Successful Aging. Am J Public Health, 1994. 84(11): p. 1796–9. doi: 10.2105/ajph.84.11.1796 7977920 PMC1615223

[44] Rogers J.C., et al., Stability and change in functional assessment of patients with geropsychiatric disorders. Am J Occup Ther, 1994. 48(10): p. 914–8. doi: 10.5014/ajot.48.10.914 7825707

[45] Blazer D.G., Hays J.C., and Foley D.J., Sleep complaints in older adults: a racial comparison. J Gerontol A Biol Sci Med Sci, 1995. 50(5): p. M280–4. doi: 10.1093/gerona/50a.5.m280 7671031

[46] McCallum J., Shadbolt B., and Wang D., Self-rated health and survival: a 7-year follow-up study of Australian elderly. Am J Public Health, 1994. 84(7): p. 1100–5. doi: 10.2105/ajph.84.7.1100 8017532 PMC1614762

[47] Forsell Y. and Winblad B., Feelings of anxiety and associated variables in a very elderly population. Int J Geriatr Psychiatry, 1998. 13(7): p. 454–8. 9695033 10.1002/(sici)1099-1166(199807)13:7<454::aid-gps795>3.0.co;2-d

[48] Andresen E.M., et al., Screening for depression in well older adults: evaluation of a short form of the CES-D (Center for Epidemiologic Studies Depression Scale). Am J Prev Med, 1994. 10(2): p. 77–84. 8037935

[49] Boey K.W., Cross-validation of a short form of the CES-D in Chinese elderly. Int J Geriatr Psychiatry, 1999. 14(8): p. 608–17. 10489651 10.1002/(sici)1099-1166(199908)14:8<608::aid-gps991>3.0.co;2-z

[50] Baron E.C., Davies T., and Lund C., Validation of the 10-item Centre for Epidemiological Studies Depression Scale (CES-D-10) in Zulu, Xhosa and Afrikaans populations in South Africa. BMC Psychiatry, 2017. 17(1): p. 6. doi: 10.1186/s12888-016-1178-x 28068955 PMC5223549

[51] Sherbourne C.D. and Stewart A.L., The MOS social support survey. Soc Sci Med, 1991. 32(6): p. 705–14. doi: 10.1016/0277-9536(91)90150-b 2035047

[52] Agresti A., *Categorical Data Analysis*. 3rd Edition ed. 2012: John Wiley & Sons Inc.

[53] Altman D., Bland MJ, Statistics Notes—Interaction revisited: the difference between two estimates. BMJ, 2003. 326: p. 219. doi: 10.1136/bmj.326.7382.219 12543843 PMC1125071

[54] Schafer I., et al., Multimorbidity patterns in the elderly: a new approach of disease clustering identifies complex interrelations between chronic conditions. PLoS One, 2010. 5(12): p. e15941. doi: 10.1371/journal.pone.0015941 21209965 PMC3012106

[55] Hair J., Black WC, Babin BJ, Anderson RE, *Multivariate Data Analysis*. 7th Edition ed. 2009, Upper Saddle River, 761: Pearson Prentice Hall.

[56] Stubbs B., et al., Depression and physical health multimorbidity: primary data and country-wide meta-analysis of population data from 190 593 people across 43 low- and middle-income countries. Psychol Med, 2017. 47(12): p. 2107–2117. doi: 10.1017/S0033291717000551 28374652

[57] Ho C., et al., Coexisting medical comorbidity and depression: multiplicative effects on health outcomes in older adults. Int Psychogeriatr, 2014. 26(7): p. 1221–9. doi: 10.1017/S1041610214000611 24735786

[58] Byers A.L., et al., High occurrence of mood and anxiety disorders among older adults: The National Comorbidity Survey Replication. Arch Gen Psychiatry, 2010. 67(5): p. 489–96. doi: 10.1001/archgenpsychiatry.2010.35 20439830 PMC2933177

[59] Goldberg D., The detection and treatment of depression in the physically ill. World Psychiatry, 2010. 9(1): p. 16–20. doi: 10.1002/j.2051-5545.2010.tb00256.x 20148148 PMC2816927

[60] Garin N., et al., Multimorbidity patterns in a national representative sample of the Spanish adult population. PLoS One, 2014. 9(1): p. e84794. doi: 10.1371/journal.pone.0084794 24465433 PMC3896355

[61] Gunn J.M., et al., The association between chronic illness, multimorbidity and depressive symptoms in an Australian primary care cohort. Soc Psychiatry Psychiatr Epidemiol, 2012. 47(2): p. 175–84. doi: 10.1007/s00127-010-0330-z 21184214

[62] Fiest K.M., et al., Chronic conditions and major depression in community-dwelling older adults. J Affect Disord, 2011. 131(1–3): p. 172–8. doi: 10.1016/j.jad.2010.11.028 21168918

[63] Temple J.B., et al., Discrimination reported by older adults living with mental health conditions: types, contexts and association with healthcare barriers. Soc Psychiatry Psychiatr Epidemiol, 2020. doi: 10.1007/s00127-020-01914-9 32696302

[64] Prados-Torres A., et al., Multimorbidity patterns: a systematic review. J Clin Epidemiol, 2014. 67(3): p. 254–66. doi: 10.1016/j.jclinepi.2013.09.021 24472295

[65] Prados-Torres A., et al., Multimorbidity patterns in primary care: interactions among chronic diseases using factor analysis. PLoS One, 2012. 7(2): p. e32190. doi: 10.1371/journal.pone.0032190 22393389 PMC3290548

[66] Haug T.T., Mykletun A., and Dahl A.A., Are anxiety and depression related to gastrointestinal symptoms in the general population? Scand J Gastroenterol, 2002. 37(3): p. 294–8. doi: 10.1080/003655202317284192 11916191

[67] Newcomer S.R., Steiner J.F., and Bayliss E.A., Identifying subgroups of complex patients with cluster analysis. Am J Manag Care, 2011. 17(8): p. e324–32. 21851140

[68] Garcia-Olmos L., et al., Comorbidity patterns in patients with chronic diseases in general practice. PLoS One, 2012. 7(2): p. e32141. doi: 10.1371/journal.pone.0032141 22359665 PMC3281110

[69] Yavuz B.G., et al., Association between somatic amplification, anxiety, depression, stress and migraine. J Headache Pain, 2013. 14: p. 53. doi: 10.1186/1129-2377-14-53 23799958 PMC3695888

[70] Jette N., Amoozegar F., and Patten S.B., Depression in epilepsy, migraine, and multiple sclerosis: Epidemiology and how to screen for it. Neurol Clin Pract, 2017. 7(2): p. 118–127. doi: 10.1212/CPJ.0000000000000349 29185533 PMC5669406

[71] Haug T.T., Mykletun A., and Dahl A.A., The association between anxiety, depression, and somatic symptoms in a large population: the HUNT-II study. Psychosom Med, 2004. 66(6): p. 845–51. doi: 10.1097/01.psy.0000145823.85658.0c 15564348

[72] van der Feltz-Cornelis C.M. and van Balkom A.J., The concept of comorbidity in somatoform disorder—a DSM-V alternative for the DSM-IV classification of somatoform disorder. J Psychosom Res, 2010. 68(1): p. 97–9; author reply 99–100. doi: 10.1016/j.jpsychores.2009.09.011 20004307

[73] van der Leeuw G., et al., The association between somatization and disability in primary care patients. J Psychosom Res, 2015. 79(2): p. 117–22. doi: 10.1016/j.jpsychores.2015.03.001 25824596

[74] Hoedeman R., et al., The contribution of high levels of somatic symptom severity to sickness absence duration, disability and discharge. J Occup Rehabil, 2010. 20(2): p. 264–73. doi: 10.1007/s10926-010-9239-3 20373134 PMC2887510

[75] Verhaak P.F., et al., Depression, disability and somatic diseases among elderly. J Affect Disord, 2014. 167: p. 187–91. doi: 10.1016/j.jad.2014.05.057 24992026

[76] Canadian Institute for Health Information, Seniors And The Health Care System: What Is The Impact Of Multiple Chronic Conditions?. 2011, CIHI Ottawa, Ontario.

[77] Griffith L.E., et al., Insights on multimorbidity and associated health service use and costs from three population-based studies of older adults in Ontario with diabetes, dementia and stroke. BMC Health Serv Res, 2019. 19(1): p. 313. doi: 10.1186/s12913-019-4149-3 31096989 PMC6524233

