# Supplementary figures and images for: Functional limitations in people with multimorbidity and the association with mental health conditions: Baseline data from the Canadian Longitudinal Study on Aging (CLSA)
Source: PLoS One. 2021 Aug 11;16(8):e0255907. doi: 10.1371/journal.pone.0255907 (PMC8357170; doi:10.1371/journal.pone.0255907)

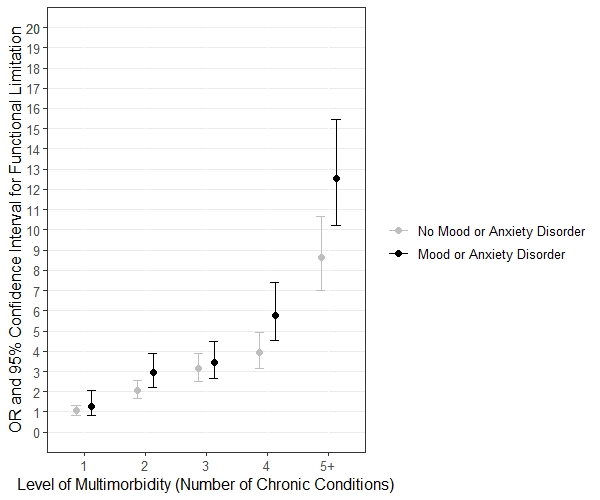

Supplement: S1 Fig — (JPEG) [file pone.0255907.s001.jpeg]

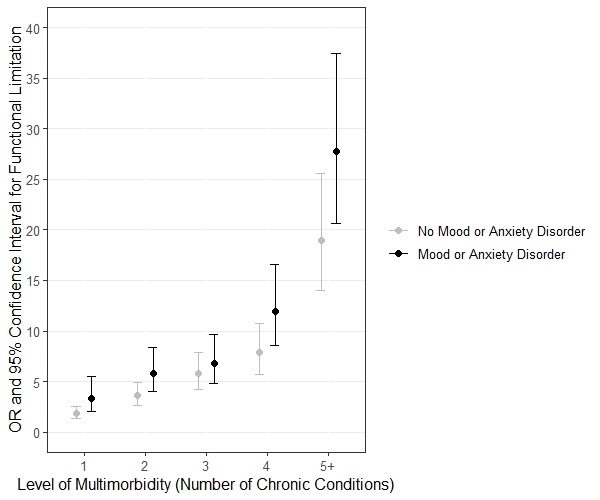

Supplement: S2 Fig — (JPEG) [file pone.0255907.s002.jpeg]
