# Supplementary material for: Functional limitations in people with multimorbidity and the association with mental health conditions: Baseline data from the Canadian Longitudinal Study on Aging (CLSA)
Source: PLoS One. 2021 Aug 11;16(8):e0255907. doi: 10.1371/journal.pone.0255907 (PMC8357170; doi:10.1371/journal.pone.0255907)
Supplement: S1 File — Additional information about the Canadian Longitudinal Study on Aging. (DOCX) [file pone.0255907.s003.docx]

**S1 File**

The CLSA is a national stratified sample of over 50,000 women and men aged 45-85 years of age at the time of recruitment. It is one of the most comprehensive research platforms in the world, and is collecting a wide range of information about the changing biological, medical, psychological, social, lifestyle and economic aspects of people’s lives. In 2015, the CLSA completed recruitment and baseline data collection on community-living women and men aged 45 to 85 years from across Canada. Three sampling frames were used for CLSA recruitment: 1) recruitment from a subset of participant’s in the Statistics Canada’s Canadian Community Health Survey – Healthy Aging (CCHS-HA), which is a nationally representative sample of Canadians > 45 years of age; 2) recruitment from provincial health care system registries; and 3) recruitment using Random Digit Dialing (RDD) of landline phones. Similar to the CCHS-HA, people were excluded if they lived on federal First Nations reserves, were full-time members of the Canadian Armed Forces, lived in institutions, were unable to respond in English or French, or were cognitively impaired. Sample size calculations for the CLSA were difficult to establish due to the diversity of research studies (both foreseen and yet unknown); however, simulations based on projected evolutions of the cohort experience over time were used (similar to the UK Biobank strategy) as well as the adequacy of power profiles for two outcomes (hazard ratios, odds ratios). These analyses suggested the adequacy of an overall sample size of 50,000, with 30,000 for the comprehensive cohort (see below).

Of the 51,338 CLSA participants enrolled at baseline, 21,241 provided information through telephone interviews (CLSA Tracking) and 30,097 through in-home interviews (CLSA Comprehensive). In addition to the in-home interview, CLSA Comprehensive participants also visited one of 11 data collection sites (DCS) across Canada to undergo detailed physical assessments and to provide blood and urine samples. Participants will undergo repeated waves of data collection at three-year intervals for at least 20 years. To maximize retention, during the first wave of data collection a mid-wave, maintaining contact questionnaire (MCQ) was implemented for all participants via telephone to update contact information and collect a small amount of additional data. This eighteen-month follow-up was completed in January 2016, with a retention rate of over 97%.

The CLSA is a research platform, thus the sample size was chosen to meet a diversity of analytic goals. The validity and reliability of all measures captured in the CLSA questionnaires, as well as references, can be found on the data portal od the CLSA website ([www.clas-elcv.ca](http://www.clas-elcv.ca)).

The analysis presented in this paper uses cross-sectional baseline questionnaire data from all 51,338 CLSA participants.
