# Supplementary material for: Functional limitations in people with multimorbidity and the association with mental health conditions: Baseline data from the Canadian Longitudinal Study on Aging (CLSA)
Source: PLoS One. 2021 Aug 11;16(8):e0255907. doi: 10.1371/journal.pone.0255907 (PMC8357170; doi:10.1371/journal.pone.0255907)
Supplement: S2 File — (DOCX) [file pone.0255907.s004.docx]

**S2 File**

***Contingency Table of Frequencies for Figure 1 (All) Variables***

| **Level of Multimorbidity** | **Mood/Anxiety Disorder** | **Any Functional Limitation** | | **Total** |
| --- | --- | --- | --- | --- |
|  |  | **Yes** | **No** |  |
| **1** | **Yes** | 34 | 1063 | 1097 |
|  | **No** | 255 | 9151 | 9406 |
| **2** | **Yes** | 131 | 1686 | 1817 |
|  | **No** | 481 | 7986 | 8467 |
| **3** | **Yes** | 179 | 1734 | 1913 |
|  | **No** | 625 | 5667 | 6292 |
| **4** | **Yes** | 252 | 1391 | 1643 |
|  | **No** | 560 | 3621 | 4181 |
| **5+** | **Yes** | 1136 | 2356 | 3492 |
|  | **No** | 1366 | 3617 | 4983 |

***Log-linear Model Results for Figure 1 (All) Variables***

***(FL=Functional Limitation, MM = Level of Multimorbidity, Mood=Mood/Anxiety Disorders)***

| **Model #** | **Loglinear Model** | **Deviance (G^2^)** | **df** | **P-value** | **AIC** |
| --- | --- | --- | --- | --- | --- |
| 0 | Complete Independence  (FL+MM+Mood) | 6313.7 | 13 | << 0.05 | 6502.3 |
| Models with 1 Two-Factor Interaction Term | | | | | |
| 1a | Block Independence  (MMMood+FL) | 3559.3 | 9 | << 0.05 | 3756 |
| 1b | Block Independence  (MMFL + Mood) | 2790.8 | 9 | << 0.05 | 2987.4 |
| 1c | Block Independence  (MM + MoodFL) | 5923.4 | 12 | << 0.05 | 6114.1 |
| Models with 2 Two-Factor Interaction Terms | | | | | |
| 2a | Partial Independence  (MMmMood + MMFL) | 36.426 | 5 | < 0.05  (=0.00912) | 241.05 |
| 2b | Partial Independence  (MMMood + MoodFL) | 3169.1 | 8 | << 0.05 | 3367.7 |
| 2c | Partial Independence  (MMFL + MoodFL) | 2400.5 | 8 | << 0.05 | 2599.1 |
| Model with 3 Two-Factor Interaction Terms | | | | | |
| **3^a^** | **Uniform Association = Homogeneous Association**  **(MMMood + MMFL + MoodFL)** | **10.337** | **4** | **< 0.05**  **(=0.03511)** | **216.96** |
| Model with 3 Two-Factor Terms & 1 Three-Factor Interaction Term | | | | | |
| **4^a^** | **Fully Saturated**  **(MMMood + MMFL + MoodFL + MMMoodFL** | **0.00** | **0** | **1.00** | **214.62** |

^a^ Model 4 (fully saturated model) shows acceptable fit with data, although the homogeneous model is very close when comparing the diagnostic results for the two models. The diagnostic results for Model 4 are equivalent to a logistic model with FL as the dependent variable and MM, Mood and MMxMood (interaction term) as independent variables. The diagnostic results for Model 3 (homogeneous model) are equivalent to a logistic model with FL as the dependent variable and MM and Mood (and non interaction term) as the independent variables.

***Odds Ratios & 95% Confidence Intervals (Homogeneous Association Model) – Figure 1 (All)***

***(MM=Level of Multimorbidity, Mood = Mood/Anxiety Disorder, FL = Functional Limitation)***

| **Variable Values** | **Reference** | **Odds Ratio (95% CI)** |
| --- | --- | --- |
| **MM = 2** | | |
| Mood = Yes | MM = 1 | 1.83 (1.69-1.98) |
| FL = Yes | MM = 1 | 2.21 (1.92-2.55) |
| Mood = Yes, FL = Yes | Mood = No | 1.19 (1.12-1.28) |
| **MM = 3** | | |
| Mood = Yes | MM = 1 | 2.57 (2.37-2.79) |
| FL = Yes | MM = 1 | 3.75 (3.27–4.31) |
| Mood = Yes, FL = Yes | Mood = No | 1.19 (1.12-1.28) |
| **MM = 4** | | |
| Mood = Yes | MM = 1 | 3.30 (3.03-3.59) |
| FL = Yes | MM = 1 | 5.54 (4.83, 6.38) |
| Mood = Yes, FL = Yes | Mood = No | 1.19 (1.12-1.28) |
| **MM = 5+** | | |
| Mood = Yes | MM = 1 | 5.73 (5.30-6.20) |
| FL = Yes | MM = 1 | 14.02 (12.36-15.95) |
| Mood = Yes, FL = Yes | Mood = No | 1.19 (1.12-1.28) |
