# Supplementary material for: Functional limitations in people with multimorbidity and the association with mental health conditions: Baseline data from the Canadian Longitudinal Study on Aging (CLSA)
Source: PLoS One. 2021 Aug 11;16(8):e0255907. doi: 10.1371/journal.pone.0255907 (PMC8357170; doi:10.1371/journal.pone.0255907)
Supplement: S3 File — (DOCX) [file pone.0255907.s005.docx]

**S3 File**

***Contingency Table of Frequencies for Figure 1A (Women) Variables***

| **Level of Multimorbidity** | **Mood/Anxiety Disorder** | **Any Functional Limitation** | | **Total** |
| --- | --- | --- | --- | --- |
|  |  | **Yes** | **No** |  |
| **1** | **Yes** | 25 | 530 | 555 |
|  | **No** | 149 | 3928 | 4077 |
| **2** | **Yes** | 101 | 938 | 1039 |
|  | **No** | 305 | 3703 | 4008 |
| **3** | **Yes** | 129 | 1048 | 1177 |
|  | **No** | 425 | 2863 | 3288 |
| **4** | **Yes** | 193 | 833 | 1026 |
|  | **No** | 375 | 1896 | 2271 |
| **5+** | **Yes** | 894 | 1575 | 2469 |
|  | **No** | 961 | 2028 | 2989 |

***Log-linear Model Results for Figure 1A (Women) Variables***

***(FL=Functional Limitation, MM = Level of Multimorbidity, Mood=Mood/Anxiety Disorders)***

| **Model #** | **Loglinear Model** | **Deviance (G^2^)** | **df** | **P-value** | **AIC** |
| --- | --- | --- | --- | --- | --- |
| 0 | Complete Independence  (FL+MM+Mood) | 3708.3 | 13 | << 0.05 | 3887.2 |
| Models with 1 Two-Factor Interaction Term | | | | | |
| 1a | Block Independence  (MMMood+FL) | 2116.2 | 9 | << 0.05 | 2303 |
| 1b | Block Independence  (MMFL + Mood) | 1613.5 | 9 | << 0.05 | 2800.3 |
| 1c | Block Independence  (MM + MoodFL) | 3492.4 | 12 | << 0.05 | 3673.2 |
| Models with 2 Two-Factor Interaction Terms | | | | | |
| 2a | Partial Independence  (MMmMood + MMFL) | 21.336 | 5 | < 0.05  (0.0007) | 216.2 |
| 2b | Partial Independence  (MMMood + MoodFL) | 1900.2 | 8 | << 0.05 | 2089.1 |
| 2c | Partial Independence  (MMFL + MoodFL) | 1397.5 | 8 | << 0.05 | 1586.4 |
| Model with 3 Two-Factor Interaction Terms | | | | | |
| **3^a^** | **Uniform Association = Homogeneous Association**  **(MMMood + MMFL + MoodFL)** | **11.5** | **4** | **< 0.05**  **(0.02)** | **208.37** |
| Model with 3 Two-Factor Terms & 1 Three-Factor Interaction Term | | | | | |
| 4 | Fully Saturated  (MMMood + MMFL + MoodFL + MMMoodFL | 0.00 | 0 | 1.00 | 214.62 |

^a^ Model 3 (homogeneous association model) shows the best overall fit with data when comparing all the diagnostic criteria. The diagnostic results for Model 3 are equivalent to a logistic model with FL as the dependent variable and MM and Mood (and no interaction term) as the independent variables.

***Odds Ratios & 95% Confidence Intervals (Homogeneous Association Model) – Figure 1A (Women) (MM=Level of Multimorbidity, Mood = Mood/Anxiety Disorder, FL = Functional Limitation)***

| **Variable Values** | **Reference** | **Odds Ratio (95% CI)** |
| --- | --- | --- |
| **MM = 2** | | |
| Mood = Yes | MM = 1 | 1.89 (1.69-2.11) |
| FL = Yes | MM = 1 | 2.22 (1.85-2.66) |
| Mood = Yes, FL = Yes | Mood = No | 1.14 (1.05-1.23) |
| **MM = 3** | | |
| Mood = Yes | MM = 1 | 2.60 (2.33-2.91) |
| FL = Yes | MM = 1 | 3.56 (2.99-4.26) |
| Mood = Yes, FL = Yes | Mood = No | 1.14 (1.05-1.23) |
| **MM = 4** | | |
| Mood = Yes | MM = 1 | 3.26 (2.91-3.66) |
| FL = Yes | MM = 1 | 5.20 (4.37-6.22) |
| Mood = Yes, FL = Yes | Mood = No | 1.14 (1.05-1.23) |
| **MM = 5+** | | |
| Mood = Yes | MM = 1 | 5.84 (5.25-6.49) |
| FL = Yes | MM = 1 | 12.68 (10.76-14.93) |
| Mood = Yes, FL = Yes | Mood = No | 1.14 (1.05-1.23) |
