# Supplementary material for: Functional limitations in people with multimorbidity and the association with mental health conditions: Baseline data from the Canadian Longitudinal Study on Aging (CLSA)
Source: PLoS One. 2021 Aug 11;16(8):e0255907. doi: 10.1371/journal.pone.0255907 (PMC8357170; doi:10.1371/journal.pone.0255907)
Supplement: S4 File — (DOCX) [file pone.0255907.s006.docx]

**S4 File**

***Contingency Table of Frequencies for Figure 1B (Men) Variables***

| **Level of Multimorbidity** | **Mood/Anxiety Disorder** | **Any Functional Limitation** | |  |
| --- | --- | --- | --- | --- |
|  |  | **Yes** | **No** | **Total** |
| **1** | **Yes** | 9 | 533 | 542 |
|  | **No** | 106 | 5223 | 5329 |
| **2** | **Yes** | 30 | 748 | 778 |
|  | **No** | 176 | 4283 | 4459 |
| **3** | **Yes** | 50 | 686 | 736 |
|  | **No** | 200 | 2804 | 3004 |
| **4** | **Yes** | 59 | 558 | 617 |
|  | **No** | 185 | 1725 | 1910 |
| **5+** | **Yes** | 242 | 781 | 1023 |
|  | **No** | 405 | 1589 | 1994 |

***Log-linear Model Results for Figure 1B (Men) Variables***

***(FL=Functional Limitation, MM = Level of Multimorbidity, Mood=Mood/Anxiety Disorders)***

| **Model #** | **Loglinear Model** | **Deviance (G^2^)** | **df** | **P-value** | **AIC** |
| --- | --- | --- | --- | --- | --- |
| 0 | Complete Independence  (FL+MM+Mood) | 1984.6 | 13 | <0.05 | 2152.9 |
| Models with 1 Two-Factor Interaction Terms | | | | | |
| 1a | Block Independence  (MMMood+FL) | 1081.5 | 9 | <0.05 | 1257.8 |
| 1b | Block Independence  (MMFL + Mood) | 907.87 | 9 | <0.05 | 1084.2 |
| 1c | Block Independence  (MM + MoodFL) | 1914 | 12 | <0.05 | 2084.3 |
| Model with 2 Two-Factor Interaction Terms | | | | | |
| **2a** | **Conditional Independence**  **(MMMood + MMFL)** | **4.7708** | **5** | **0.44** | **189.1** |

^a^ Model 2a (conditional independence) shows acceptable fit with data. The diagnostic results for this model are equivalent to a logistic model with FL as the dependent variable and MM as the only independent variable.

***Odds Ratios & 95% Confidence Intervals (Conditional Independence Model 2a) - Figure 1B (Men) (MM=Level of Multimorbidity, Mood = Mood/Anxiety Disorder, FL = Functional Limitation)***

| **Variable Values** | **Reference** | **Odds Ratio (95% CI)** |
| --- | --- | --- |
| **MM = 2** | | |
| Mood = Yes | MM = 1 | 1.72 (1.53-1.93) |
| FL = Yes | MM = 1 | 2.05 (1.63-2.59) |
| **MM = 3** | | |
| Mood = Yes | MM = 1 | 2.41 (2.14-2.72) |
| FL = Yes | MM = 1 | 3.59 (2.87-4.50) |
| **MM = 4** | | |
| Mood = Yes | MM = 1 | 3.18 (2.80-3.61) |
| FL = Yes | MM = 1 | 5.35 (4.27-6.73) |
| **MM = 5+** | | |
| Mood = Yes | MM = 1 | 5.04 (4.40-5.67) |
| FL = Yes | MM = 1 | 13.66 (11.19-16.83) |
