# Supplementary material for: Functional limitations in people with multimorbidity and the association with mental health conditions: Baseline data from the Canadian Longitudinal Study on Aging (CLSA)
Source: PLoS One. 2021 Aug 11;16(8):e0255907. doi: 10.1371/journal.pone.0255907 (PMC8357170; doi:10.1371/journal.pone.0255907)
Supplement: S5 File — (DOCX) [file pone.0255907.s007.docx]

**S5 File**

***Contingency Table of Frequencies for Figure 2 Variables***

|  |  | **Any ADL/IADL** | |
| --- | --- | --- | --- |
| **MM** | **AGE** | **Yes** | **No** |
| **0** | **45-54** | **59** | **3821** |
|  | **55-64** | **48** | **2569** |
|  | **65-74** | **16** | **791** |
|  | **75+** | **16** | **237** |
| **1** | **45-54** | **80** | **3916** |
|  | **55-64** | **80** | **3710** |
|  | **65-74** | **62** | **1839** |
|  | **75+** | **73** | **784** |
| **2** | **45-54** | **120** | **2468** |
|  | **55-64** | **211** | **3477** |
|  | **65-74** | **121** | **2388** |
|  | **75+** | **160** | **1361** |
| **3** | **45-54** | **117** | **1328** |
|  | **55-64** | **206** | **2403** |
|  | **65-74** | **192** | **2121** |
|  | **75+** | **295** | **1557** |
| **4** | **45-54** | **100** | **632** |
|  | **55-64** | **210** | **1431** |
|  | **65-74** | **190** | **1557** |
|  | **75+** | **316** | **1401** |
| **5+** | **45-54** | **213** | **507** |
|  | **55-64** | **515** | **1454** |
|  | **65-74** | **635** | **1992** |
|  | **75+** | **1142** | **2027** |

***Log-linear Model Results for Figure 2 Variables***

***(FL=Functional Limitation, MM = Level of Multimorbidity, Mood=Mood/Anxiety Disorders)***

| **Model #** | **Loglinear Model** | **Deviance (G^2^)** | **df** | **P-value** | **AIC** |
| --- | --- | --- | --- | --- | --- |
| 0 | Complete Independence  (FL+MM+Age) | 14.387 | 38 | << 0.05 | 14790 |
| Models with 1 Two-Factor Interaction Terms | | | | | |
| 1a | Block Independence  (MMAge+FL) | 4863.5 | 23 | << 0.05 | 5296.4 |
| 1b | Block Independence  (MMFL + Age) | 9935.4 | 33 | << 0.05 | 10348 |
| 1c | Block Independence  (MM + AgeFL) | 12826 | 35 | << 0.05 | 13235 |
| Models with 2 Two-Factor Interaction Terms | | | | | |
| 2a | Partial Independence  (MMAge + MMFL) | 411.53 | 18 | < 0.05 | 854.47 |
| 2b | Partial Independence  (MMAge + AgeFL) | 3302.3 | 20 | << 0.05 | 3741.2 |
| 2c | Partial Independence  (MMFL + AgeFL) | 8374.2 | 30 | << 0.05 | 8793.1 |
| Model with 3 Two-Factor Interaction Terms | | | | | |
| 3 | Uniform Association = Homogeneous Association  (MMAge + MMFL + AgeFL) | 75.549 | 15 | << 0.05 | 524.49 |
| Model with 3 Two-Factor Interactions and 1 Three-Factor Interaction | | | | | |
| **4** | **Fully Saturated**  **(MMAge+MMFL+**  **AgeFL + MMAgeFL** | **0** | **0** | **1.00** | **478.94** |

^a^ Model 4 (fully saturated model) shows acceptable fit with data, The diagnostic results for Model 4 are equivalent to a logistic model with FL as the dependent variable and MM, Age and MMxAge (interaction term) as independent variables.

***Odds Ratios & 95% Confidence Intervals (Homogeneous Association Model) – Figure 2***

***(MM=Level of Multimorbidity, Mood = Mood/Anxiety Disorder, FL = Functional Limitation)***

| **Variable Values** | **Reference** | **Odds Ratio (95% CI)** |
| --- | --- | --- |
| **MM = 1** | | |
| Age = 55-64 | MM = 0 | 1.41 (1.32-1.51) |
| Age = 65-74 | MM = 0 | 2.27 (2.06-2.49) |
| Age=75+ | MM = 0 | 3.23 (2.78-3.76) |
| FL = Yes | MM = 0 | 1.32 (0.94-1.86) |
| Age = 55-64, FL = Yes | Age = 45-54 | 1.21 (0.82-1.77) |
| Age = 65-74, FL = Yes | Age = 45-54 | 1.31 (0.73-2.23) |
| Age = 75+, FL = Yes | Age = 45-54 | 4.37 (2.40-7.53) |
| **MM = 3** | | |
| Age = 55-64 | MM = 0 | 2.69 (2.48-2.93) |
| Age = 65-74 | MM = 0 | 7.72(6.96-8.55) |
| Age=75+ | MM = 0 | 18.90 (16.30-22.01) |
| FL = Yes | MM = 0 | 5.71 (4.17-7.90) |
| Age = 55-64, FL = Yes | Age = 45-54 | 1.21 (0.82-1.77) |
| Age = 65-74, FL = Yes | Age = 45-54 | 1.31 (0.73-2.23) |
| Age = 75+, FL = Yes | Age = 45-54 | 4.37 (2.40-7.53) |
| **MM = 4** | | |
| Age = 55-64 | MM = 0 | 3.37 (3.03-3.75) |
| Age = 65-74 | MM = 0 | 11.90 (10.56-13.43) |
| Age=75+ | MM = 0 | 35.74 (30.47-42.08) |
| FL = Yes | MM = 0 | 10.25 (7.37-14.36) |
| Age = 55-64, FL = Yes | Age = 45-54 | 1.21 (0.82-1.77) |
| Age = 65-74, FL = Yes | Age = 45-54 | 1.31 (0.73-2.23) |
| Age = 75+, FL = Yes | Age = 45-54 | 4.37 (2.40-7.53) |
| **MM = 5+** | | |
| Age = 55-64 | MM = 0 | 4.27 (3.81-4.78) |
| Age = 65-74 | MM = 0 | 18.98 (16.78-21.50) |
| Age=75+ | MM = 0 | 64.46 (54.85-76.05) |
| FL = Yes | MM = 0 | 27.21 (20.23-37.12) |
| Age = 55-64, FL = Yes | Age = 45-54 | 1.21 (0.82-1.77) |
| Age = 65-74, FL = Yes | Age = 45-54 | 1.31 (0.73-2.23) |
| Age = 75+, FL = Yes | Age = 45-54 | 4.37 (2.40-7.53) |
