# Supplementary material for: Functional limitations in people with multimorbidity and the association with mental health conditions: Baseline data from the Canadian Longitudinal Study on Aging (CLSA)
Source: PLoS One. 2021 Aug 11;16(8):e0255907. doi: 10.1371/journal.pone.0255907 (PMC8357170; doi:10.1371/journal.pone.0255907)
Supplement: S6 File — (DOCX) [file pone.0255907.s008.docx]

**S6 File**

***SEX***

***Contingency Table of Frequencies***

| **Level of Multimorbidity** | **Sex** | **Any Functional Limitation** | |  |
| --- | --- | --- | --- | --- |
|  |  | **Yes** | **No** | **Total** |
| **1** | **F** | 174 | 4458 | 4632 |
|  | **M** | 115 | 5756 | 5871 |
| **2** | **F** | 406 | 4641 | 5047 |
|  | **M** | 206 | 5031 | 5237 |
| **3** | **F** | 554 | 3911 | 4465 |
|  | **M** | 250 | 3490 | 3740 |
| **4** | **F** | 568 | 2729 | 3297 |
|  | **M** | 244 | 2283 | 2527 |
| **5+** | **F** | 1855 | 3603 | 5458 |
|  | **M** | 647 | 2370 | 3017 |

***Log-linear Model Results***

***(FL=Functional Limitation, MM = Level of Multimorbidity, Mood=Mood/Anxiety Disorders)***

| **Model #** | **Loglinear Model** | **Deviance (G^2^)** | **df** | **P-value** | **AIC** |
| --- | --- | --- | --- | --- | --- |
| 0 | Complete Independence  (anyadl+MM+sex) | 4816.7 | 13 | <<0.05 | 5008.4 |
|  | Models with 1 Two-Factor Interaction Term | | | | |
| 1a | Block Independence  (MMsex+anyadl) | 3932.6 | 9 | <<0.05 | 4132.2 |
| 1b | Block Independence  (MManyadl + sex) | 1293.8 | 9 | <<0.05 | 1493.5 |
| 1c | Block Independence  (MM + sexanyadl) | 4055.7 | 12 | <<0.05 | 4249.4 |
| Models with 2 Two-Factor Interactions Terms | | | | | |
| 2a | Partial Independence  (MMsex + MManyadl) | 409.64 | 5 | <0.05 | 617.38 |
| 2b | Partial Independence  (MMsex + sexanyadl) | 3171.5 | 8 | <<0.05 | 3373.3 |
| 2c | Partial Independence  (MManyadl + sexanyadl) | 4055.7 | 12 | <<0.05 | 4249.4 |
| Model with 3 Two-Factor Interaction Terms | | | | | |
| **3^a^** | **Uniform Association = Homogeneous Association**  **(MMsex + MManyadl + sexanyadl)** | **1.5142** | **4** | **0.82** | **211.25** |

^a^ Model 3 (homogeneous association model) shows acceptable fit with the data. The diagnostic results for Model 3 are equivalent to a logistic model with FL as the dependent variable and MM and Sex (and no interaction term) as the independent variables.

***INCOME***

***Contingency Table of Frequencies***

| **Level of Multimorbidity** | **Income** | **Any Functional Limitation** | | **Total** |
| --- | --- | --- | --- | --- |
|  |  | **Yes** | **No** |  |
| **0** | **1 = <$20k** | 10 | 162 | 172 |
|  | **2 = $20k - $49.9k** | 29 | 989 | 1018 |
|  | **3 = $50k - $99.9k** | 46 | 2414 | 2460 |
|  | **4 = $100k - $149.9k** | 27 | 1781 | 1808 |
|  | **5 = $150k+** | 19 | 1737 | 1756 |
| **1** | **1 = <$20k** | 29 | 321 | 350 |
|  | **2 = $20k - $49.9k** | 93 | 1755 | 1848 |
|  | **3 = $50k - $99.9k** | 91 | 3442 | 3533 |
|  | **4 = $100k - $149.9k** | 39 | 2184 | 2223 |
|  | **5 = $150k+** | 16 | 2002 | 2018 |
| **2** | **1 = <$20k** | 64 | 388 | 452 |
|  | **2 = $20k - $49.9k** | 181 | 2117 | 2298 |
|  | **3 = $50k - $99.9k** | 203 | 3464 | 3667 |
|  | **4 = $100k - $149.9k** | 78 | 1761 | 1839 |
|  | **5 = $150k+** | 40 | 1432 | 1472 |
| **3** | **1 = <$20k** | 95 | 398 | 493 |
|  | **2 = $20k - $49.9k** | 268 | 1893 | 2161 |
|  | **3 = $50k - $99.9k** | 232 | 2588 | 2820 |
|  | **4 = $100k - $149.9k** | 82 | 1234 | 1316 |
|  | **5 = $150k+** | 48 | 800 | 848 |
| **4** | **1 = <$20k** | 120 | 300 | 420 |
|  | **2 = $20k - $49.9k** | 269 | 1449 | 1718 |
|  | **3 = $50k - $99.9k** | 235 | 1757 | 1992 |
|  | **4 = $100k - $149.9k** | 79 | 697 | 776 |
|  | **5 = $150k+** | 29 | 452 | 481 |
| **5+** | **1 = <$20k** | 444 | 532 | 976 |
|  | **2 = $20k - $49.9k** | 1047 | 2000 | 3047 |
|  | **3 = $50k - $99.9k** | 599 | 1955 | 2554 |
|  | **4 = $100k - $149.9k** | 120 | 618 | 738 |
|  | **5 = $150k+** | 78 | 368 | 446 |

***Log-linear Model Results***

***(FL=Functional Limitation, MM = Level of Multimorbidity, Mood=Mood/Anxiety Disorders)***

| **Model #** | **Loglinear Model** | **Logistic Model** | **Deviance (G^2^)** | **df** | **P-value** | **AIC** | **BIC^a^** |
| --- | --- | --- | --- | --- | --- | --- | --- |
| 0 | Complete Independence  (anyadl+MM+inc) |  | 9043.1 | 49 | <<0.05 | 9518.8 |  |
| Models with 1 Two-Factor Interaction Term | | | | | | | |
| 1a | Block Independence  (MMinc+anyadl) | Null | 4904.6 | 29 | <<0.05 | 5420.3 |  |
| 1b | Block Independence  (MManyadl + inc) |  | 4877.3 | 44 | <<0.05 | 5363 |  |
| 1c | Block Independence  (MM + incanyadl) |  | 7210.6 | 45 | <<0.05 | 7694.3 |  |
| Models with 2 Two-Factor Interaction Terms | | | | | | | |
| 2a | Partial Independence  (MMinc + MManyadl) | MM | 738.86 | 24 | <<0.05 | 1264.6 |  |
| 2b | Partial Independence  (MMinc + educanyadl) | Inc | 3072.1 | 25 | <<0.05 | 3595.8 |  |
| 2c | Partial Independence  (MManyadl + incanyadl) |  | 3044.8 | 40 | <<0.05 | 3538.5 |  |
| Model with 3 Two-Factor Interaction Terms | | | | | | | |
| **3^a^** | **Uniform Association = Homogeneous Association**  **(MMinc + MManyadl + incanyadl)** | **MM+ Inc** | **30.089** | **20** | **0.07** | **563.81** |  |

^a^ Model 3 (homogeneous association model) shows acceptable fit with the data. The diagnostic results for Model 3 are equivalent to a logistic model with FL as the dependent variable and MM and Income (and no interaction term) as the independent variables.

***EDUCATION***

***Contingency Table of Frequencies***

| **MM** | **EDUCATION** | **Any ADL/IADL** | |  |
| --- | --- | --- | --- | --- |
|  |  | **Yes** | **No** | **Total** |
| **0** | **1= No High School** | 9 | 221 | 230 |
|  | **2= High School** | 18 | 714 | 732 |
|  | **3 = Some University** | 11 | 433 | 444 |
|  | **4 = University or Graduate School** | 100 | 6039 | 6139 |
| **1** | **1= No High School** | 27 | 440 | 467 |
|  | **2= High School** | 36 | 1091 | 1127 |
|  | **3 = Some University** | 24 | 671 | 695 |
|  | **4 = University or Graduate School** | 204 | 8029 | 8233 |
| **2** | **1= No High School** | 62 | 572 | 634 |
|  | **2= High School** | 81 | 1034 | 1115 |
|  | **3 = Some University** | 56 | 712 | 768 |
|  | **4 = University or Graduate School** | 407 | 7354 | 7761 |
| **3** | **1= No High School** | 87 | 553 | 640 |
|  | **2= High School** | 94 | 832 | 926 |
|  | **3 = Some University** | 67 | 597 | 664 |
|  | **4 = University or Graduate School** | 559 | 5409 | 5968 |
| **4** | **1= No High School** | 102 | 438 | 540 |
|  | **2= High School** | 115 | 596 | 711 |
|  | **3 = Some University** | 83 | 401 | 484 |
|  | **4 = University or Graduate School** | 512 | 3575 | 4087 |
| **5+** | **1= No High School** | 388 | 681 | 1069 |
|  | **2= High School** | 313 | 751 | 1064 |
|  | **3 = Some University** | 248 | 526 | 774 |
|  | **4 = University or Graduate School** | 1547 | 3997 | 5544 |

***Log-linear Model Results***

***(FL=Functional Limitation, MM = Level of Multimorbidity, Educ = Education Level^a^)***

| **Model #** | **Loglinear Model** | **Deviance (G^2^)** | **df** | **P-value** | **AIC** |
| --- | --- | --- | --- | --- | --- |
| 0 | Complete Independence  (anyadl+MM+educ) | 5563.9 | 38 | <<0.05 | 5948.8 |
| Model with 1 Two-Factor Interaction Term | | | | | |
| 1a | Block Independence  (MMeduc+anyadl) | 4574.9 | 23 | <<0.05 | 4989.8 |
| 1b | Block Independence  (MManyadl + educ) | 1107.1 | 33 | <<0.05 | 1502 |
| 1c | Block Independence  (MM + educanyadl) | 5204.9 | 35 | <<0.05 | 5595.9 |
| Model with 2 Two-Factor Interaction Terms | | | | | |
| 2a | Partial Independence  (MMeduc + MManyadl) | 118.11 | 18 | <<0.05 | 543.04 |
| 2b | Partial Independence  (MMeduc + educanyadl) | 4215.9 | 20 | <<0.05 | 4636.9 |
| 2c | Partial Independence  (MManyadl + educanyadl) | 748.15 | 30 | <<0.05 | 1149.1 |
| Model with 3 Two-Factor Interaction Terms | | | | | |
| **3^a^** | **Uniform Association = Homogeneous Association**  **(MMeduc + MManyadl + educanyadl)** | **15.748** | **15** | **0.40** | 446.68 |

^a^ Model 3 (homogeneous association model) shows acceptable fit with the data. The diagnostic results for Model 3 are equivalent to a logistic model with FL as the dependent variable and MM and Education (and no interaction term) as the independent variables.

***LIVING ARRANGEMENT***

***Contingency Table of Frequencies***

| **Level of Multimorbidity** | **Live Arrangement**  **(Living Alone – Yes/No)** | **Any Functional Limitation** | | |
| --- | --- | --- | --- | --- |
|  |  | **Yes** | **No** | **Total** |
| **0** | **Yes** | 28 | 111 | 139 |
|  | **No** | 945 | 6473 | 7418 |
| **1** | **Yes** | 89 | 206 | 295 |
|  | **No** | 1704 | 8545 | 10249 |
| **2** | **Yes** | 162 | 450 | 612 |
|  | **No** | 1946 | 7748 | 9694 |
| **3** | **Yes** | 276 | 534 | 810 |
|  | **No** | 1845 | 5564 | 7409 |
| **4** | **Yes** | 293 | 523 | 816 |
|  | **No** | 1327 | 3694 | 5021 |
| **5+** | **Yes** | 1066 | 1439 | 2505 |
|  | **No** | 1942 | 4038 | 5980 |

***Log-linear Model Results***

***(FL=Functional Limitation, MM = Level of Multimorbidity, Alone = Living Arrangement)***

| **Model #** | **Loglinear Model** | **Deviance (G^2^)** | **df** | **P-value** | **AIC** |
| --- | --- | --- | --- | --- | --- |
| 0 | Complete Independence  (FL+MM+Alone) | 6183.6 | 16 | <<0.05 | 6406.9 |
| **Models with 1 Two-Factor Interaction Term** | | | | | |
| 1a | Block Independence  (MMAlone+FL) | 1731.6 | 11 | <<0.05 | 1964.9 |
| 1b | Block Independence  (MML + Alone) | 4641.2 | 11 | <<0.05 | 4874.6 |
| 1c | Block Independence  (MM + AloneFL) | 5590 | 15 | <<0.05 | 5815.4 |
| **Models with 2 Two-Factor Interaction Terms** | | | | | |
| 2a | Partial Independence  (MMAlone+ MMFL) | 189.25 | 6 | <0.05 | 432.58 |
| 2b | Partial Independence  (MMAlone + AloneFL) | 1138.1 | 10 | <<0.05 | 1373.4 |
| 2c | Partial Independence  (MMFL + AloneFL) | 4047.7 | 10 | <<0.05 | 4283 |
| **Model with 3 Two-Factor Interaction Terms** | | | | | |
| **3^a^** | **Uniform Association = Homogeneous Association**  **(MMAlone + MMFL + AloneFL)** | **7.2156** | **5** | **0.21** | **252.55** |

^a^ Model 3 (homogeneous association model) shows acceptable fit with the data. The diagnostic results for Model 3 are equivalent to a logistic model with FL as the dependent variable and MM and Living Arrangement (and no interaction term) as the independent variables.

***SOCIAL SUPPORT***

***Contingency Table of Frequencies***

| **Level of Multimorbidity** | **Social Support Quartile** | **Any Functional Limitation** | |  |
| --- | --- | --- | --- | --- |
|  |  | **Yes** | **No** | **Total** |
| **0** | **1** | 37 | 1487 | 1524 |
|  | **2** | 32 | 1800 | 1832 |
|  | **3** | 31 | 1831 | 1862 |
|  | **4** | 35 | 2163 | 2198 |
| **1** | **1** | 88 | 2125 | 2213 |
|  | **2** | 81 | 2492 | 2573 |
|  | **3** | 58 | 2546 | 2604 |
|  | **4** | 55 | 2818 | 2873 |
| **2** | **1** | 184 | 2309 | 2493 |
|  | **2** | 143 | 2353 | 2496 |
|  | **3** | 140 | 2341 | 2481 |
|  | **4** | 118 | 2413 | 2531 |
| **3** | **1** | 249 | 1858 | 2107 |
|  | **2** | 189 | 1800 | 1989 |
|  | **3** | 158 | 1760 | 1918 |
|  | **4** | 156 | 1720 | 1876 |
| **4** | **1** | 287 | 1336 | 1623 |
|  | **2** | 181 | 1214 | 1395 |
|  | **3** | 166 | 1200 | 1366 |
|  | **4** | 134 | 1069 | 1203 |
| **5+** | **1** | 1005 | 1786 | 2791 |
|  | **2** | 539 | 1437 | 1976 |
|  | **3** | 490 | 1341 | 1831 |
|  | **4** | 313 | 1147 | 1460 |

***Log-linear Model Results***

***(FL=Functional Limitation, MM = Level of Multimorbidity, Socsup = Social Support)***

| **Model #** | **Loglinear Model** | **Deviance (G^2^)** | **df** | **P-value** | **AIC** |
| --- | --- | --- | --- | --- | --- |
| 0 | Complete Independence  (FL+MM+Socsup) | 5145.5 | 38 | <<0.05 | 5551.2 |
| **Models with 1 Two-Factor Interaction Term** | | | | | |
| 1a | Block Independence  (MMSocsup+FL) | 4425.2 | 23 | <<0.05 | 4860.9 |
| 1b | Block Independence  (MMFL + Socsup) | 929.04 | 33 | <<0.05 | 1344.7 |
| 1c | Block Independence  (MM + SocsupFL) | 4694.2 | 35 | <<0.05 | 5105.9 |
| **Models with 2 Two-Factor Interaction Terms** | | | | | |
| 2a | Partial Independence  (MMSocsup + MMFL) | 208.72 | 18 | <0.05 | 654.39 |
| 2b | Partial Independence  (MMSocsup + SocsupFL) | 3973.9 | 20 | <<0.05 | 4415.5 |
| 2c | Partial Independence  (MMFL + SocsupFL) | 477.72 | 30 | <<0.05 | 899.39 |
| **Model with 3 Two-Factor Interaction Terms** | | | | | |
| **3^a^** | **Uniform Association = Homogeneous Association**  **(MMSocsup + MMFL + SocsupFL)** | **13.686** | **15** | **0.55** | **465.36** |

^a^ Model 3 (homogeneous association model) shows acceptable fit with data. The diagnostic results for Model 3 are equivalent to a logistic model with FL as the dependent variable and MM and Social Support (and non interaction term) as the independent variables.
